# Tailoring MXene Thickness and Functionalization for Enhanced Room-Temperature Trace NO_2_ Sensing

**DOI:** 10.1007/s40820-023-01316-x

**Published:** 2024-01-12

**Authors:** Muhammad Hilal, Woochul Yang, Yongha Hwang, Wanfeng Xie

**Affiliations:** 1 Department of Physics, Dongguk University, Seoul, 04620 Republic of Korea; 2 Department of Control and Instrumentation Engineering, Korea University, Sejong, 30019 Republic of Korea; 3 School of Electronics & Information, University- Industry Joint Center for Ocean Observation and Broadband Communication, Qingdao University, Qingdao, 266071 China

**Keywords:** Controlled MXene thickness, Gaseous functionalization approach, Lower electronegativity functional groups, Enhanced MXene stability, Trace NO_2_ sensing

## Abstract

In this study, precise control over the thickness and termination of Ti_3_C_2_T_X_ MXene flakes is achieved to enhance their electrical properties, environmental stability, and gas-sensing performance. Utilizing a hybrid method involving high-pressure processing, stirring, and immiscible solutions, sub-100 nm MXene flake thickness is achieved within the MXene film on the Si-wafer. Functionalization control is achieved by defunctionalizing MXene at 650 °C under vacuum and H_2_ gas in a CVD furnace, followed by refunctionalization with iodine and bromine vaporization from a bubbler attached to the CVD. Notably, the introduction of iodine, which has a larger atomic size, lower electronegativity, reduce shielding effect, and lower hydrophilicity (contact angle: 99°), profoundly affecting MXene. It improves the surface area (36.2 cm^2^ g^−1^), oxidation stability in aqueous/ambient environments (21 days/80 days), and film conductivity (749 S m^−1^). Additionally, it significantly enhances the gas-sensing performance, including the sensitivity (0.1119 Ω ppm^−1^), response (0.2% and 23% to 50 ppb and 200 ppm NO_2_), and response/recovery times (90/100 s). The reduced shielding effect of the –I-terminals and the metallic characteristics of MXene enhance the selectivity of I-MXene toward NO_2_. This approach paves the way for the development of stable and high-performance gas-sensing two-dimensional materials with promising prospects for future studies.

**Supplementary Information:**

The online version contains supplementary material available at 10.1007/s40820-023-01316-x.

## Highlights

- Gas-phase functionalization of X-MXene (X = –F, –OH, –O, –Br, –I) films crafted from sub-100 nm thin MXene flakes for highly sensitive NO_2_ sensors.
- I-MXene-based senor exhibited significant sensing performances toward trace NO_2_ at room temperature.
- The hydrophobicity, larger atomic size, lower electronegativity, and reduced shielding of -I contribute to the excellent sensing enhancement of I-MXene.

**Supplementary Information:**

The online version contains supplementary material available at 10.1007/s40820-023-01316-x.

## Graphical abstract

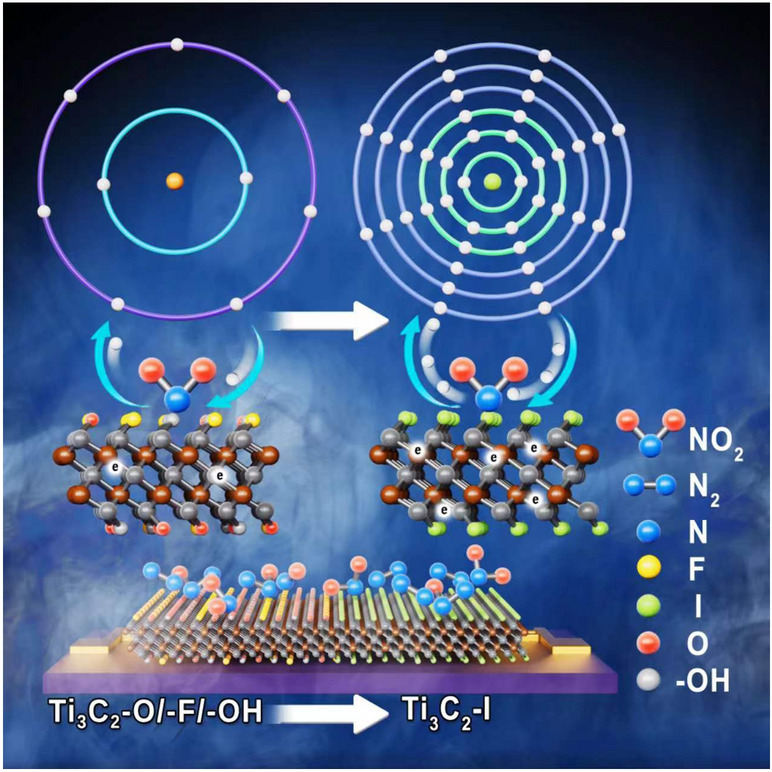

## Introduction

In recent years, interest in 2D materials, especially MXenes, has grown due to their unique properties and applications in electronics, energy storage, and sensors [1–3]. MXenes, derived from MAX phases, exhibit exceptional properties, including high electrical conductivity, large surface area, and tailorable functional groups [4, 5]. The key to obtaining high-quality MXenes lies in the selective etching of the A-layer, resulting in surface functional groups such as –O, –OH, and –F [6].

Since the discovery of MXene, hydrofluoric acid (HF) has remained the most commonly employed etching method because of its exceptional selectivity in etching the A-layer from the MAX phases. Naguib et al. played a pioneering role in developing this method, marking a significant milestone in the field [7]. However, owing to the hazardous nature of HF, researchers explored alternative etchants, like lithium fluoride (LiF), sodium fluoride (NaF), potassium fluoride (KF), and ammonium fluoride (NH_4_F) salts in hydrochloric acid (HCl) [8]. Although this approach mitigates the direct use of HF, it is essential to note that it still carries inherent risks, as the reaction with HCl can lead to the release of toxic HF gas. Consequently, efforts focused on more safer approaches. Halim et al. substituted HF with NH_4_HF_2_ [9], while Libo et al. employed a hydrothermal method with NH_4_F (pressurization technique) [10]. Xie et al. utilized NaOH and hydrothermal treatments [11], Aihu et al. employed NH_4_HF_2_ [12], and Biao et al. used water-assisted potassium hydroxide for MXene production [13].

Despite much progress in etching methods, achieving successful MXene preparation remains a challenge, especially for researchers without a material or chemical background. Existing methods may require multiple attempts, making it uncertain to obtain high-quality products. One limitation of the stirred-based methods is the incomplete penetration of the etchant between the stacked layers, which can result in residual Al. Similarly, the autoclave treatments may suffer from incomplete etching due to a lack of stirring. To address these issues, a new approach combining simultaneous stirring and pressurizing is needed for effective etching. This ensures proper interaction with each flake and the production of high-quality MXenes. Furthermore, Precise control over MXene thickness and size is crucial for enhancing their properties, including electrical conductivity, optical characteristics, surface catalysis and overall performances [14–17]. This control is often achieved through exfoliation processes, such as tip-sonication of MXene in various exfoliating solvents, such as dimethyl sulfoxide (DMSO) and *N*-methyl-2-pyrrolidone (NMP) [18]. However, it's important to note that exfoliation is a secondary process in MXene synthesis, with etching being the primary factor that determines thickness and size control. Skillful etching allows successful exfoliation, resulting in MXenes with various thicknesses and sizes to meet specific application requirements.

To further improve properties of MXene or based materials, such as electrical conductivity, optical characteristics, and stability, control over surface functional groups (–O, –OH, –F) is crucial [14, 19, 20]. While defunctionalization can improve conductivity [14], the ambient reattachment of –O and –OH groups degrade their stability and performance. To improve the characteristics of MXenes, various approaches have been proposed, including incorporating metal oxides, defect control, polymer composites, and optimizing storage environments [2, 21–24]. However, studies on the surface engineering of effective protective layers that enhance the characteristics of MXenes is still limited. Previous studies have explored surface engineering using solution-based techniques. For instance, functionalizing MXenes with fluoroalkylsilane (FOTS) molecules resulted in a superhydrophobic surface, improved stability, and enhanced gas-sensing performance [25]. Similarly, Jingjing et al. functionalized MXenes with (3-Aminopropyl)triethoxysilane for efficient stabilization [26]. Lim et al. utilized alkylsilane coupling agents to increase the surface hydrophobicity of Ti_3_C_2_T_*x*_ MXenes, enabling their dispersibility in nonpolar solvents such as hexane [27]. Similarly, Xin et al. introduced hydrocarbon termination to reduce the hydrophilicity of Ti_3_C_2_T_*x*_ MXene and improve its sensitivity to volatile organic compounds [28]. Prior work improved MXene stability and sensing performance despite unwanted functional groups (–O/–OH and –F), which negatively impact conductivity and overall performance, hindering comprehensive understanding of new functional groups' effects. This emphasizes the need for a cleaning step before introducing desired functional groups to study their impact. Current solution-based methods have limitations, necessitating a liquid-free technique to remove unwanted functional groups and graft target ones. This approach could substantially enhance MXene properties and performance for broader applications, such as gas sensing performances.

Previous MXene research mainly utilized molecular functional groups [21, 22, 24], limiting direct comparison with elemental MXene functional groups (–O/–OH and –F) in terms of fundamental characteristics (electronegativity, atomic size, shielding effect, and hydrophilicity) [25, 26, 28]. Investigating the elemental forms of these functional groups is essential for comprehensive understanding. Some studies explored elemental functional groups to enhance MXene properties. For example, Shi et al. enhanced ambient stability by replacing –F with –I [29], and Wang et al. effectively tuned the work function of Ti_3_C_2_T_*x*_ MXene using oxygen plasma treatment to enhance electron transport in perovskite solar cells [30]. Although halogenated functional groups (such as –I, –Br, and –IBr) [31, 32] have been studied in energy storage, their role in gas sensing is underexplored. Additionally, MXenes possess inherent metallic properties, allowing efficient electron donation to adsorbed gas molecules, regardless of whether they are oxidizing or reducing gases [25, 33]. However, the high electronegativity and shielding effect of –O/–OH and –F result in prolonged response and recovery times. Also, the smaller atomic size of –O/–OH and –F reduces interlayer space and specific surface area. Therefore, exploring substitutions of –O/–OH and –F functional groups with other elements provides insights into their effects on MXene properties in gas sensing.

Herein, we implemented a comprehensive approach to achieve precise control over the thickness of MXene flakes within the MXene film. Additionally, we developed a unique gas-phase method, conducted in an isolated environment, to defunctionalize and functionalize MXenes with elemental forms of functional groups (–I and –Br). This research sheds light on the fundamental characteristics of these functional groups and their interplay with the metallic nature of MXenes, particularly in gas-sensing applications. We found that iodine, serving as a hydrophobic terminal (contact angle: 99°) on the Ti_3_C_2_ MXene surface, effectively counteracts oxidative instability. Furthermore, the larger atomic size of iodine, lower electronegativity, and lower shielding effect significantly increased the specific surface area of MXene (36.2 cm^2^ g^−1^), conductivity (749 S m^−1^), sensitivity (0.1119 Ω ppm^−1^), linear detection limit (0.05–200 ppm), and the adsorption/desorption efficiency (100/112 s). These advancements position MXenes as highly promising for advanced gas-sensing applications.

## Experimental Section

### Materials

All chemicals used in this study were obtained from various suppliers. Ti_3_AlC_2_ MAX phase was purchased from Nanochemazone (Canada). Sigma-Aldrich provided HF (48%), HCl (37%), LiF, NaF, NH_4_F, and DMSO. Iodine and Bromine were obtained from Samchun Pure Chemical Co., Ltd.

### Synthesis of Few-layer Ti_3_C_2_T_***x***_ MXene Flakes within MXene Film

**Scheme 1:**
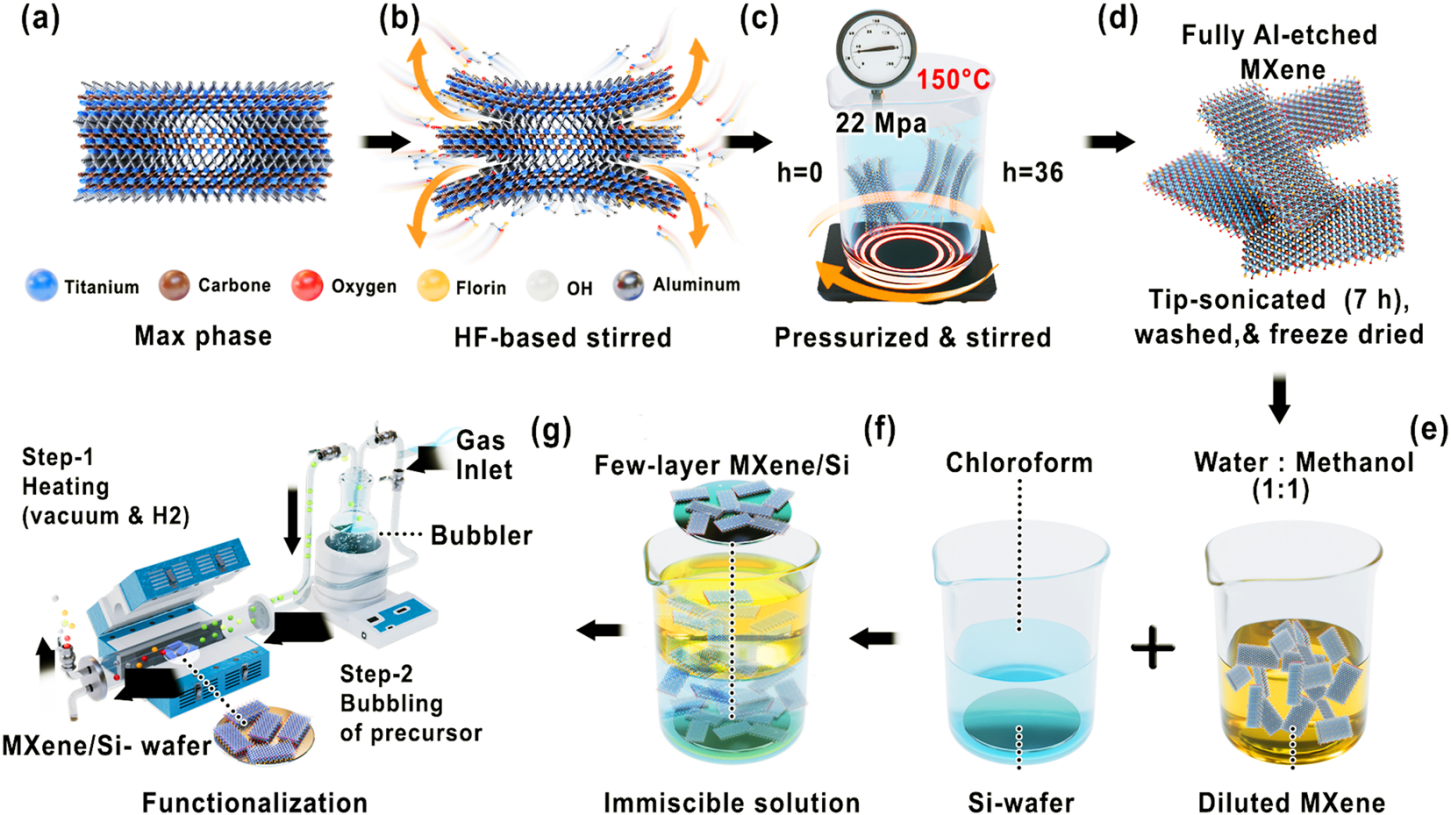
Steps involved in the **a–f** preparation and **g** functionalization of few-layer MXene

The optimized hybrid HF-Hydrothermal synthesis conditions for few-layer MXene preparation, as discussed below, were determined through a series of experimental attempts (Table SI 1–16). This involved the initial stirring of 1.0 g of Ti_3_AlC_2_ powder (Scheme 1a, b and Table S16) at 200 rpm for 36 h at room temperature in 20 mL HF (48%). The choice of etchant concentrations (HF, 48 wt% and HCl, 37 wt% as described in the Supporting Information) was based on a previous study [34], where these concentrations were found to effectively modulate MXene size. This process was followed by washing and collecting product with centrifugation using deionized (DI) water and vacuum filtration until the pH of the supernatant reached approximately 6.0.

The obtained product was transferred to a high-pressure autoclave reactor containing a 120 mL solution prepared by dissolving 5 g of NH_4_F in 120 mL of DI water. The solution was pressurized to 22 MPa for 36 h with a constant flow of Ar gas at 150 °C while stirring at 200 rpm (Scheme 1c). Subsequently, the resulting product was washed by filtration and centrifugation until the pH reached ~ 6.50 and then freeze-dried for 48 h.

**Fig. 1:**
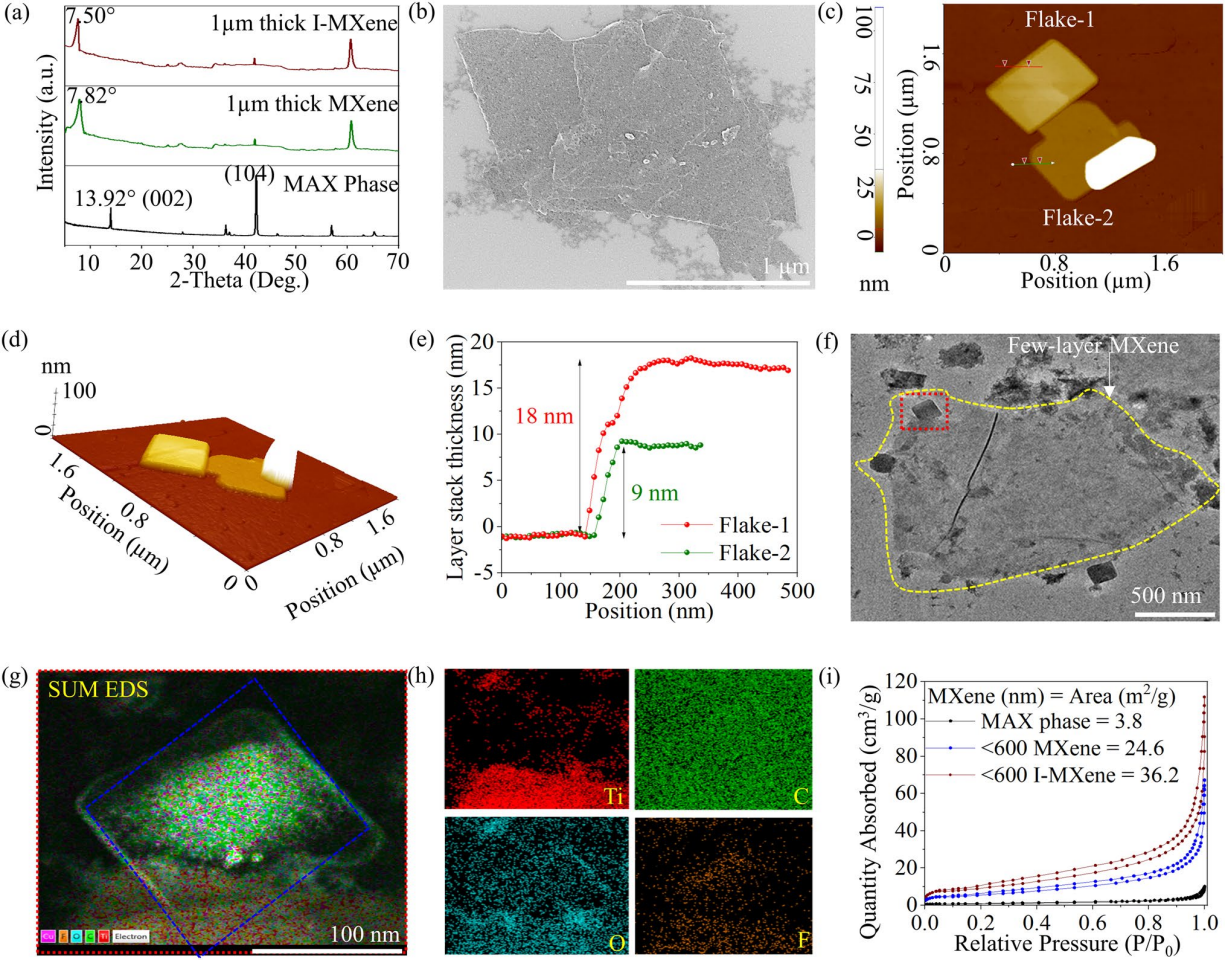
**a** XRD of MAX phase, fully etched as-prepared, and fully etched I-MXene. **b** MXene SEM image of the diluted MXene powder. **c** 2D and **d** 3D AFM images of the few-layered MXene with correspondence **e** line-profile of the thickness. **f** HRTEM image of few-layer MXene with **g** SUM EDS mapping and corresponding **h** elemental compositions. **i** BET analysis of MAX phase, few-layer as-prepared MXene, and few-layer I-MXene

The dried product was then introduced into a double-walled beaker containing DMSO and subjected to 7 h of tip sonication. The tip sonicator was equipped with a water-chiller system to maintain a constant temperature of 15 °C. Subsequently, the sample was washed by centrifugation, and the sediment was collected from two parts of the centrifuge tube (Fig. S3i), that is, bottom (flake thickness ~ 1 µm: Fig. S1j) and top (flake thickness < 100–600 nm: Fig. S5d–f), and then freeze-dried (Scheme 1d). The sediment collected from the upper part of the centrifuge tube was dispersed in water at concentrations of 5, 10, and 20 mg mL^−1^. Subsequently, these suspensions were further diluted by a factor of 10 relative to the initial sediment concentration, as illustrated in Fig. S7a [35]. The diluted MXene sample was mixed with methanol and carefully transferred dropwise into a beaker containing chloroform solvent, with a Si wafer positioned at the bottom (Scheme 1e). The immiscibility of methanol and chloroform creates an interface where few-layer MXene flakes, with stacked layers and flake thicknesses of less than 80 nm and approximately 600 nm, self-assemble (Few-layer MXene: Figs. 1c–g and S5a–.c). This assembly results in the formation of an MXene film (Fig. S8 and S9) that aligns with the substrate size on the upper surface of the chloroform solvent (Fig. S7b). Subsequently, excess chloroform is removed, and the film level is adjusted to match the substrate. Finally, the substrate is lifted and subjected to a drying process at 100 °C for approximately 30 min to ensure complete dryness of the film (Scheme 1f). Notably, achieving few-layer MXene films is a challenge yet essential task, particularly for analyzing gas sensing performance. Traditional methods like spin-coating or drop-casting often result in non-uniform and thick films, limiting sensing capabilities. In contrast, the immiscible approach for film formation, validated by SEM (Fig. S8) and TEM (Fig. S9) analysis, offers distinct advantages. This technique surpasses other methods, such as spin-coating (Fig. S5d) and drop-casting (Fig. S16c), by consistently producing films composed of few-layer MXene flakes on a scale Si-wafer. This enhances the specific surface area and active sites available for gas adsorption.

### Functionalization of Few-Layer Ti_3_C_2_T_***x***_ MXene

For the precise surface modification of few-layer MXenes, a gas-phase approach utilizing a CVD furnace with a bubbler system was employed (Scheme 1g). The obtained few-layer MXene film on the Si-wafer underwent heating at 650 °C under vacuum and H_2_ gas environment for 2 h in each case. This thermal treatment effectively eliminated –O, –F, and –OH functional groups, resulting in a clean MXene surface. The integrated bubbler system in the CVD furnace contained I– and Br– as the desired termination elements. Vaporized iodine was introduced into the furnace using Ar gas flow at 50 °C for 30 min, selectively attaching to the clean surface of MXene. Similarly, Bromine vapor was introduced at room temperature for 30 min under Ar gas flow. The diluted MXene powder was spread over a Si wafer and subjected to a similar functionalization process, which was then characterized by Brunauer–Emmett–Teller (BET), X-ray photoelectron spectroscopy (XPS), and Fourier transform infrared (FTIR) analyses.

To fully validate the role of halogenated functional groups on electrical properties and gas sensing performances, it is essential to explore Cl-MXene as a gas sensing element. Our preferred approach for MXene functionalization involves starting with the precursor material in its elemental form. However, during the course of this study, elemental chlorine in any suitable form (liquid/solid/gas) was not available, which temporarily limited our ability to explore Cl-MXene under these specific conditions. In contrast, we explored the synthesis of Cl-MXene using a CuCl_2_ molten salt approach [32]. Unlike the high-pressure and stirring treatments involved in our as-prepared MXene synthesis, this method resulted in a reduced interlayer separation, as shown in Fig. S6a, b. Due to the minimal interlayer spacing, Cl-MXene exhibited higher resistance and poorer gas-sensing performance compared to our as-prepared MXene (Fig. S21a, b). Additionally, the Cl-MXene was not transformed into few-layer MXene to compare its role with few-layer I- and Br-MXene. As preparation of few-layer from multi-layer Cl-MXene, need to underwent prolonged exfoliation process. This exfoliation process would effectively alter its functional group composition, leading to changes in its properties and performance. As a result, our chosen functionalization approach involves first preparing MXene with fewer stacked layers and then functionalizing it in a controlled gaseous phase environment, allowing for the versatile production of few-layer MXene with various functional groups. Further details regarding Cl-MXene synthesis, characteristics, and gas-sensing performances are discussed in the Supporting Information.

### Characterization

The surface morphologies, microstructures, and crystallinities of different Ti_3_C_2_T_*x*_ MXene products were analyzed using various characterization techniques. Scanning electron microscopy (SEM) was performed using a JEOL-7800F instrument, whereas transmission electron microscopy (TEM) was performed using a JEM-ARM 200F instrument (NEOARM) equipped with a high-angle annular dark-field scanning electron microscope (HAADF-STEM) and energy-dispersive spectrometer (EDS). The thicknesses of the few-layer MXene samples were determined by atomic force microscopy (AFM, Model: NX-10, Park System). X-ray diffraction (XRD) measurements were performed using an Ultima IV instrument (Rigaku, Japan). The chemical components and bonding structures resulting from the iodine and bromine functionalization were investigated using X-ray photoelectron spectroscopy (XPS (mono) (Model: K-alpha)) and FTIR analysis (FT-IR Spectrometer, Model: Cary670). BET analysis was performed using an Autosorb IQ instrument to determine the specific surface area of each prepared sample. The optical absorbance was measured using a V-750 UV–visible spectrofluorometer equipped with a diffuse reflectance and fluorescence detector (JASCO FP-8600).

### Gas Sensing Device Preparation and Measurement Process

In the preparation of gas sensing devices, two different scenarios were used based on the MXene film preparation and functionalization mythology. Firstly, we considered two MXene samples, including as-prepared MXene flakes-with stacked layers and flake thicknesses of approximately 100 nm and 10 µm (highly etched as-prepared MXene) and -with stacked layers and flake thicknesses ranging from < 100 to 600 nm and approximately 1 µm (fully etched as-prepared MXene). Both samples underwent a common device preparation process, which included drop-casting from a 2 mg mL^−1^ stack solution onto interdigitated gold (Au) electrodes coated over a Si-wafer. (Fig. S16c). To ensure the quality of the interdigitated electrode structure, we used the shadow mask pattern with length, width, and spacing of 8.641, 3.281, and 0.188 mm, respectively (Fig. S16a). The optical image of the device (Fig. S16b) clearly shows that MXene was drop-casted onto the Au-electrode pattern, ensuring the ohmic contacts, as MXene behaves also like a metal. This is further confirmed by the ohmic response obtained in the I–V curve (Fig. S16d). The completed sensor devices were then placed within a 5 cm^3^ chamber and connected to the signal acquisition system via slender gold wires using silver paste (Fig. S16e).

On the other hand, we produced the few-layer MXene film using an immiscible solution approach with a tenfold diluted sample of 10 mg mL^−1^ MXene sediment from the upper part of a centrifuge tube (Fig. S3i). SEM and TEM images of this MXene-based film (obtained among films prepared via the tenfold dilution of 5, 10, and 20 mg mL^−1^) are provided in Figs. S8 and S9. This film was then functionalized with I- and Br-. Subsequently, the same process of thermal evaporation and gold wire soldering, with silver paste, was applied to establish a connection with the signal acquisition system.

To carry out the gas sensing experiments, the mass flow meters and controllers (MFC, Tylan 2900), were used to precisely dilute the target gases, including NO_2_, NH_3_, H_2_, ethanol and acetone (Deahan Gas Co., Ltd), by N_2_ gas (Daehan Gas Co., Ltd). These gases were dynamically mixed to achieve the desired gas concentrations. Prior to introducing the target gas, the chamber was evacuated to maintain vacuum conditions and minimize the influence of humidity. Electrical conductance signals of the sensors were recorded using a data acquisition system (Agilent 34970A). The response sensitivity (S%) of the sensors was calculated using the following equation:

1 $${\text{S}}\% \, = \, \Delta R/R_{{\text{a}}} \times { 1}00 \, = \, \left( {R_{{\text{g}}} - R_{{\text{a}}} } \right)/R_{{\text{a}}} \times { 1}00$$

where *R*_a_ and *R*_g_ denote the resistance of the sensors upon exposure to N_2_ and the target gases, respectively [36, 37].

## Results and Discussion

### Structural and Morphological Investigation

The XRD patterns shown in Figs. 1a and S10a reveal the partial etching of Al from the Ti_3_AlC_2_ powder. In the sample stirred in HF and exfoliated in DMSO for 7 h, we observe a slight shift of the (002) peak from 13.92° to 11.76°, accompanied by the appearance of the (104) peak at 41.92° which exhibits a 74% reduction in intensity compared to the MAX phase sample [38, 39]. These findings suggest that stirring the MAX phase in HF alone had limited success in etching Al, thus impeding its conversion into smaller MXene flakes. In contrast, through a hybrid HF-hydrothermal treatment involving pressurization of partially etched Ti_3_AlC_2_ powder within an advanced high-pressure autoclave reactor, successful etching of Al was achieved even prior to the initiation of the exfoliation process. Therefore, its XRD pattern reveals a downward shift from 11.76° to 8.84° for the (002) peak and the appearance of the (104) peak with a reduced intensity of approximately 80%. This suggests that the pressurization and stirring processes are more effective in eliminating Al content than the bare HF stirring process. Furthermore, through a prolonged tip-sonication process, the (002) peak of Ti_3_C_2_T_*x*_ experiences an additional shift from 8.84° to 7.82°, indicating an increase in interplanar distance that reduces the stacked-layer and flake thickness: < 100–600 nm and ~ 1 µm (Figs. S1j and S10a). Finally, the I-MXene XRD revealed a further downward shift of the (002) peak from 7.82° to 7.50°, indicating an increased interlayer spacing owing to steric hindrance caused by the larger iodine atoms.

SEM images in Figs. S1a, g–j and 1b illustrate the partially etched Ti_3_AlC_2_ after 7 h exfoliation (stacked-layer and flake thickness: ~ 500 nm and 10 µm prepared via HF-stirred method), highly etched Ti_3_C_2_T_*x*_ prior the exfoliation, and fully etched Ti_3_C_2_T_*x*_ after 7 h exfoliation, respectively. These images clearly demonstrate the successful transformation of bulk Ti_3_AlC_2_ to fully etched Ti_3_C_2_T_*x*_. EDS mapping of fully etched Ti_3_C_2_T_*x*_ was performed, as presented in Fig. S1j, k, confirming that C, Ti, F, and –O/–OH were its elemental constituents. Moreover, the tenfold diluted sample was spin-coated onto a Si wafer at 3000 rpm, resulting in a MXene flakes with thickness of 200–600 nm, as shown in Fig. S5e, f. The observed thickness validates the effective removal of thicker MXene flakes (fully etched MXene flakes: 1 µm) by means of dilution. Dilution enables self-assembly of sub-100 nm MXene flakes at the immiscible solvent interface on the Si substrate to form the film. Consequently, the thicknesses of the few-layer MXene on the Si wafer were measured using an AFM as 9, 18, and 63 nm, as depicted in Figs. 1c–e and S4d, e, S5b, c. Furthermore, HRTEM analysis of the few-layer MXene, was performed, along with EDS mapping that confirmed the presence of C, Ti, –F, and –O/–OH as its elemental constituents (Fig. 1f–h). The observed copper (Cu) peak originated from the Cu grid used as the substrate. Notably, the distribution of Ti appears non-uniform in the summed EDS image (Fig. 1g). This non-uniformity arises because the EDS analysis was conducted on the MXene flake enclosed within the blue dashed rectangle in Fig. 1f, where a few more MXene flakes exist at the lower edge of the analyzed flake. Consequently, the EDS analysis reflects a higher Ti content at the lower edge side than in the individual flakes analyzed. Additionally, the presence of white squares in Fig. 1c, d, and g indicated the agglomerated MXene nanoparticles, as confirmed through SEM images of fully etched MXene flakes with thickness ranging from 1 to 2 µm (Fig. S4a–c). Moreover, the AFM images of few-layer MXene (Fig.  S4d, e) further provide a comprehensive view of these MXene nanoparticles. BET analysis was performed to assess the impact of thickness and functionalization on the specific surface areas of Ti_3_C_2_T_*x*_ MXene (Figs. 1i and  S10b). The results revealed that the specific surface areas of the MAX phase, fully-etched MXene, few-layer MXene, few-layer I-MXene, and few-layer Br-MXene are 3.8, 14.9, 24.6, 31.7, and 36.2 m^2^ g^−1^, respectively, confirming successful exfoliation during the selective etching process. Notably, I-MXene exhibited a higher specific surface area of 36.2 m^2^ g^−1^, which was attributed to the increased interplanar distance resulting from the intercalation of larger iodine atoms than oxygen and fluorine, as observed in the XRD analysis. Moreover, water contact angle measurements revealed that the wettability of the Ti_3_C_2_T_*x*_ nanosheets was modified by –I and –Br terminal groups. The as-prepared Ti_3_C_2_T_*x*_ with –OH, –O, and –F functional groups exhibited a hydrophilic surface with a water contact angle (*θ*) of 39° (Fig. S11a). However, upon functionalization with bromine and iodine, the contact angle increased significantly to 77° and 99°, respectively, indicating a more hydrophobic nature (Fig. S11b, c). This hydrophobicity arose from the direct and firm interaction of the iodine and or bromine terminals with the Ti or C atoms of Ti_3_C_2_, which was achieved using a unique gas-phase approach. Owing to the firm attachment of less hydrophilic functional groups, Ti_3_C_2_ exhibits remarkable moisture protection over extended periods. For the I-MXene-based sample, we observed a substantial increase in resistance compared to the initial value in both ambient (2,412%) and aqueous (255,766%) environments on the 21^st^ and 80^th^ days, respectively (Fig. S12a–c). Notably, on the 28^th^ day, the sample (I-MXene) submerged in the aqueous medium showed no response, while the sample (I-MXene) exposed to the ambient environment displayed a resistance increase of 203,653% of the initial value on 150^th^ day. These results convincingly demonstrate the outstanding stability of I-MXene in both aqueous and ambient environments compared to the as-prepared MXene-based sample, which exhibited no response on the 21^st^ day in an aqueous medium and on the 110^th^ day in the ambient environment.

### Elemental Analysis and Functional Groups Confirmation

**Fig. 2:**
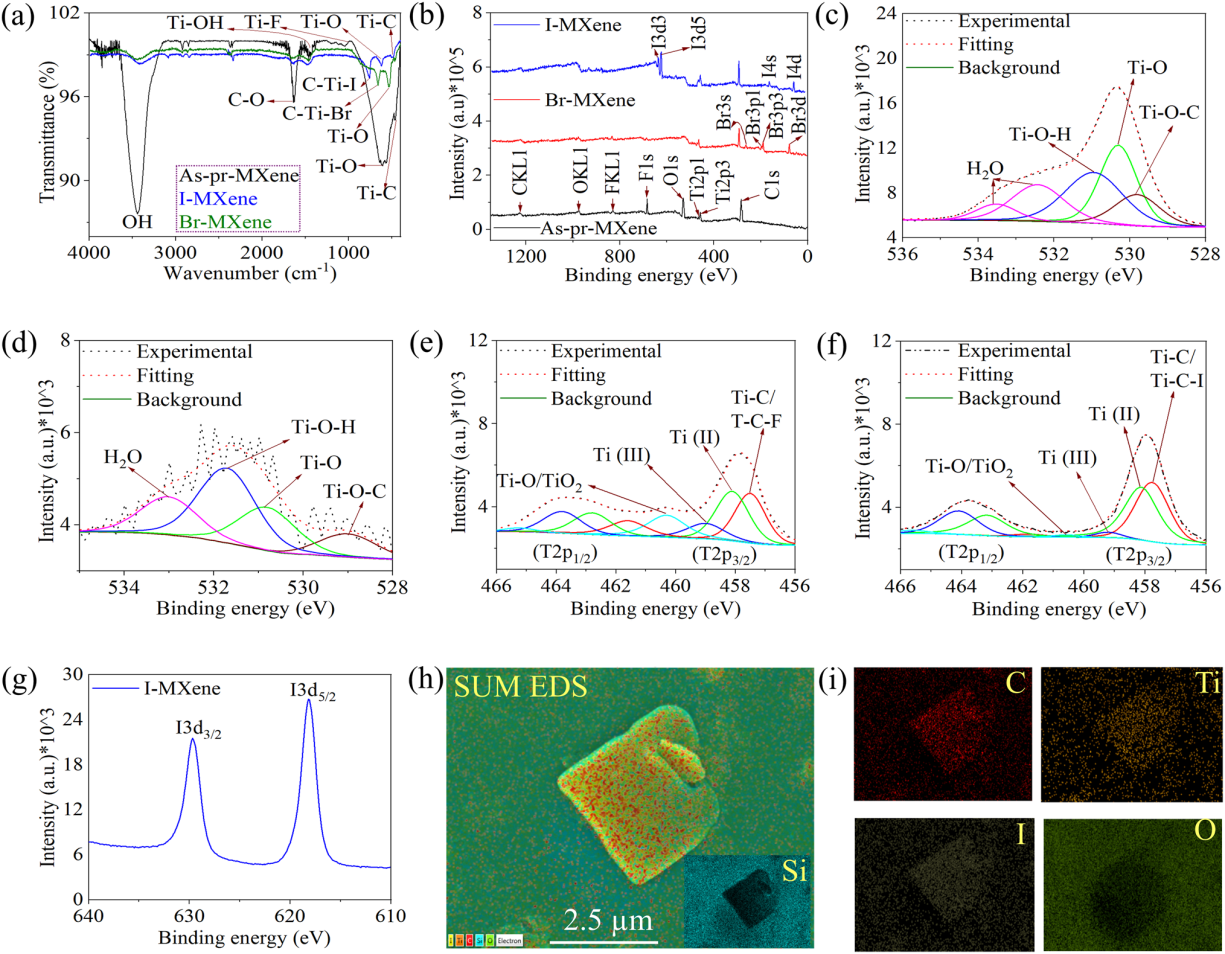
**a** FTIR and **b** XPS survey spectra of as-prepared-, I– and Br–MXene. High resolution XPS O 1*s* spectra of **c** as-prepared MXene and **d** I–MXene. Ti 2*p* spectra of **e** as-prepared MXene and **f** I–MXene. **g** I 3*d* spectrum of I–MXene. Sum EDS images of **h** I–MXene and corresponding **i** elemental composition

FTIR, XPS, and SEM with EDS analyses were performed to investigate the elemental composition and functional groups of MXene, as depicted in Figs. 2 and S13. The FTIR spectrum of the fully etched as-prepared MXene exhibited characteristic peaks corresponding to the –OH, C–O, Ti–OH, Ti–F, and Ti–O bonds at 3434, 1635, 1480, 950, and 642 cm^−1^, respectively (Fig. 2a) [26, 40, 41]. In fully etched I– and Br–MXene, a reduction in the transmittance of –OH and –F functional groups, as well as Ti–O peaks, compared to as-prepared MXene confirmed the removal of –O and –F functional groups and the attachment of Br and I functional groups, as indicated by the observed peaks at 757 cm^−1^ (C–Ti–I) and 659 cm^−1^ (C–Ti–Br) [38, 42, 43].

**Fig. 3:**
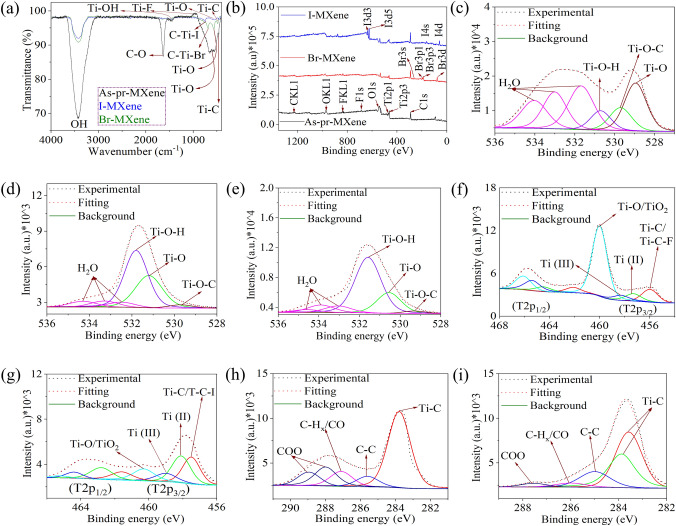
Characterization of MXene samples after one week immersion in DI water: **a** FTIR and **b** XPS survey spectra of as-prepared-, I–, and Br–MXene. **c–e** High-resolution XPS O 1*s* spectra of as-prepared, I–, and Br–MXene. Ti 2*p* spectra of **f** as-prepared-MXene and **g** I–MXene C 1*s* spectra of **h** as-prepared-MXene and **i** I–MXene

The XPS survey spectra in Fig. 2b reveal distinct peaks for the –I and –Br functional groups with minimal oxygen content and the absence of–F in I– and Br–MXene, confirming the successful elimination of the –O/–OH and –F functional groups and the introduction of –I and –Br-based functional groups. The lower intensity (at%: 7.06) of the O 1*s* spectra in I–MXene and Br–MXene (at%: 9.09%) compared to that of the as-prepared MXene (at%: 32.08%) suggests the minimal presence of water/oxygen molecules, potentially influenced by ambient environmental factors (Figs. 2c, d and S13a). In addition, the Ti 2*p* spectra of each sample (Figs. 2e, f and S13b) were fitted with four doublets of Ti 2*p*_3/2_ and Ti 2*p*_1/2_ corresponding to Ti–C, Ti^2+^, Ti^3+^, and Ti–O [25, 44]. The existence of T–O/TiO_2_ peaks with high intensity in the as-prepared MXene confirmed a higher oxygen content than those of I–MXene and Br–MXene. The C 1* s* spectra for each sample were also analyzed, revealing lower intensity peaks corresponding to C–Ti–T_*x*_, C–C, CH_x_/CO, and COO in I–MXene and Br–MXene than in the as-prepared MXene (Fig. S13c–e) [28]. Furthermore, Figs. 3g and S13f displayed the I 3*d* spectrum (at%: 23.05) of I–MXene and the Br 3*d* spectrum (at%: 21.27) of Br–MXene, confirming the successful functionalization of MXene with –I and –Br. Additionally, SEM with EDS analysis confirmed that both I and Br were successfully attached to MXene as functional groups (Figs. 2h, i and S14a–d).

### Role of Functionalization on Oxidation Stability of MXene

The oxidation stability of MXene was investigated by immersing the as-prepared I- and Br-MXene samples in DI water for one week, followed by FTIR and XPS analyses (Fig. 3). The FTIR spectra (Fig. 3a) revealed an increased transmittance of the –OH- and –O-related peaks for the immersed MXene compared to the as-prepared MXene (Fig. 2a), indicating the occurrence of oxidation. In contrast, I– and Br–MXene exhibited lower transmittances for these peaks, suggesting hindered interactions between the water molecules and Ti atoms. Similarly, the XPS survey spectrum revealed the origination of the O 1*s* peaks in both the immersed Br– and I–MXenes (Fig. 3b), indicating oxidation. The high resolution O 1*s* XPS spectra (Fig. 3c–e) further support these observations, showing significant adsorption of –O, –OH, and H_2_O molecules on the as-prepared MXene surface (at%: 64.26), leading to high-intensity peaks associated with T–O, Ti–O–C, Ti–OH, and water molecule adsorption. In contrast, I–MXene (27.18%) and Br–MXene (40.39%) demonstrated lower levels of oxidation. Ti_3_C_2_T_*x*_ MXenes undergo complete oxidation to TiO_2_ when exposed to water molecules for an extended period. To confirm this concept, the Ti_2*p*_ spectra of each sample were obtained (Figs. 3e, f and S15a), revealing a higher intensity of the Ti–O/TiO_2_ peak in the immersed as-prepared MXene, indicating the conversion of Ti (at% 13.75 in the freshly prepared MXene) to TiO_2_ (at% 8.89 in the immersed as-prepared MXene). In contrast, both I–MXene and Br–MXene retained a significant amount of Ti, as I–MXene exhibited Ti of at% of 12.8 and 9.89 in fresh I–MXene and immersed I–MXene, respectively (Fig. 3f). The Br–MXene exhibited Ti of at% of 16.51 and 12.26 in fresh Br–MXene and immersed Br–MXene, respectively (Fig. S15a). Additionally, the C 1*s* spectra exhibited higher-intensity peaks corresponding to COO and CH_*x*_/CO in the as-prepared MXene, whereas I–MXene and Br–MXene exhibited lower intensities (Figs. 3h, i and S15These findings suggest that the presence of I and Br functional groups reduces the susceptibility of MXene to oxidation because the COO and CH_*x*_/CO groups are associated with oxidation-related processes. The overall results indicate that the highly hydrophobic nature of the I and Br functional groups compared to the –O/–OH and –F functional groups effectively protects MXene from complete oxidation, contributing to improved oxidation stability.

### Role of Thickness and Functional Groups on the Gas Sensing Performance of MXene

**Fig. 4:**
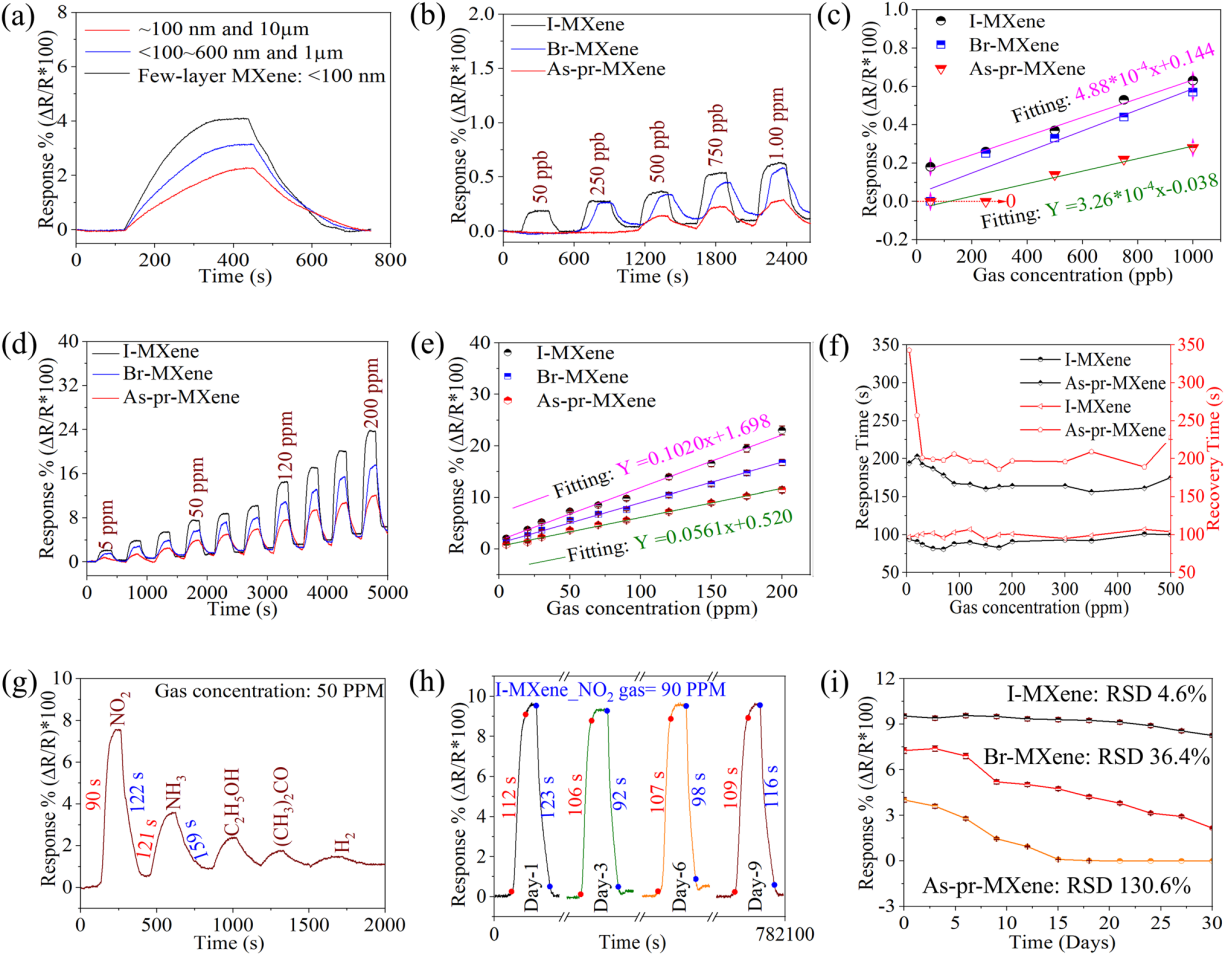
**a** Gas sensing capabilities based on different thicknesses of MXene toward 50 ppm of NO_2_. Dynamic response curves of as-prepared-, I–, and Br–MXene-based sensors to **b** lower (0.05 ppb–1 ppm) and **d** higher (5–200 ppm) NO_2_ concentrations with their corresponding **c** response versus NO_2_ concentration. **f** Sensing response and recovery time of as-prepared- and I–MXene-based sensors to NO_2_ concentrations ranging from 5 to 500 ppm. **g** Selectivity and **h** stability performances of I–MXene toward four pulses of NO_2_. **i** Long-term stability evaluation of all sensors over a 30-day period

Gas sensing experiments were conducted using Ti_3_C_2_T_*x*_ flakes of varying thicknesses and functionalizations to evaluate their impact on the gas sensing performance of MXene. For the thickness analysis, we employed three distinct sensors: highly etched as-prepared MXene, fully etched as-prepared MXene, and few-layer as-prepared MXene. To assess their gas-sensing capabilities, each sensor was exposed to NO_2_ gas at room temperature at a concentration of 50 ppm (Figs. 4a and S17). Remarkably, the few-layer MXene exhibited a significantly superior gas response of ~ 4%, outperforming the thicker flakes, which demonstrated responses of ~ 3.1% and ~ 2%, respectively (Fig. 4a). We also examined the response and recovery properties of the samples (Fig. S17). The results revealed that the few-layer MXene-based samples exhibited shorter response and recovery times of 195 and 214 s, respectively. In contrast, the fully and highly etched MXene-based sensors exhibited relatively long response/recovery times of 237/253 s and 301/297 s, respectively. The response time refers to the time required to reach 90% of the maximum response during exposure to the target gas, whereas the recovery time indicates the time taken to return to a baseline level, typically 10% of the maximum response, after the gas was removed. The enhanced gas response observed in few-layer MXene can be attributed to its high surface area (24.6 m^2^ g^−1^), providing a greater number of active sites for gas interaction. This finding highlights the significance of the thin-layered structure of MXene in improving its gas-sensing performance, making it a promising material for future gas-sensing applications. Moreover, to investigate the dose-concentration vs gas response calibration analysis, we tested different dose concentrations (5, 10, and 20 mg mL^−1^) of MXene and then diluted them tenfold for few-layer MXene film preparation using the immiscible approach. This calibration demonstrated that the tenfold diluted sample from the 10 mg mL^−1^ concentration exhibited a higher response compared to the 5 and 20 mg mL^−1^ counterparts (Fig. S19c).

Based on the exceptional gas-sensing performance of the few-layer MXene, we extended the investigation to explore its gas-sensing capabilities with I– and Br– terminations, as depicted in Fig. 4b–f. The fabricated sensors were exposed to NO_2_ gas at room temperature, covering concentrations ranging from 0.05 to 500 ppm (Figs. 4b, d and S18a). The gas-sensing responses as a function of NO_2_ concentration of all the three sensors are plotted in Figs. 4c, e and S18b, demonstrating excellent reproducibility with a relative standard deviation (RSD) of less than 5% for the average responses. In Fig. 4c, it may appear that as-prepared MXene exhibits a negative response at 250 ppb. However, the actual response of as-prepared MXene initiates at 500 ppb, with no response observed at 200 ppb. To clarify this potential confusion, we have added a red-dashed arrow in the figure, extending from zero to the point where the misunderstanding may arise. To enhance the robustness and reliability of the sensor fabrication approach using an immiscible solution, an RSD assessment was performed using two separate devices (*n* = 2) for each sensor type. Remarkably, I–MXene demonstrated outstanding responses of 0.20% and 23% toward 50 ppb and 200 ppm NO_2_, respectively, surpassing those of Br–MXene (0.23% and 17.35% for 250 ppb and 200 ppm) and the as-prepared MXene (0% and 11.38% for 250 ppb and 200 ppm) sensors. The sensitivity of a gas sensor, which is represented by the slope of its response, plays a pivotal role in determining its overall performance. For I–MXene, Br–MXene, and as-prepared MXene, the slope values within their linear detection range were determined as 0.1119 Ω ppm^−1^ (0.05 to 200 ppm), 0.0839 Ω ppm^−1^ (0.25 to 200 ppm), and 0.0603 Ω ppm^−1^ (0.50 to 200 ppm), respectively. Moreover, I–MXene exhibits significantly faster response times of ~ 90 s and recovery times of ~ 100 s (Figs. 4f and S18).

Moreover, for selectivity analysis, all the sensors were exposed to five different gases (NO_2_, NH_3_, acetone, ethanol, and H_2_) at a concentration of 50 ppm. The response plot in Figs. 4g and S19a demonstrate that both I– and Br–MXene-based sensors exhibited the highest response changes in the presence of NO_2_, indicating a high selectivity toward NO_2_ among the polar gases. Furthermore, to assess the repeatability and stability of the fabricated gas sensors during dynamic operation, we performed extensive tests by subjecting each sensor to multiple pulses of 90 ppm NO_2_ at three-day intervals for 30 days, as illustrated in Figs. 4h, i and S20a, b. The results indicated that the I–MXene-based sensor exhibited repeatable and stable dynamic responses over four consecutive cycles within the first nine days compared to Br– and the as-prepared MXene. Further analysis was performed for the full 30-day period to assess the degradation in response to each sensor. The results revealed that the I–MXene- and Br–MXene-based sensors displayed smaller changes in response, with RSD values of 4.6% and 36.4%, respectively. In contrast, the as-prepared MXene sensor exhibited no response after 15 days and had a significantly higher RSD value of 130.6%. This finding is consistent with that of our previous analysis (Fig. S12), further highlighting the excellent long-term stability and durability of I–MXene compared to those of the as-prepared MXene. Additionally, the dynamic response of the I-MXene-based sensor to 50 ppm of NO_2_ was examined across a humidity range started from vacuum-environment (dry) till 80% RH. The results, shown in Fig. S19b, indicate a response decrease from 7.4% to 1.05% with increase of humidity. This highlights the sensor's effectiveness even in high humidity, but the response reduction in humid conditions can be attributed to water molecules occupying the sensing channel's active surface, affecting its performance.

### Gas Sensing Process and I–MXene Performances

**Fig. 5:**
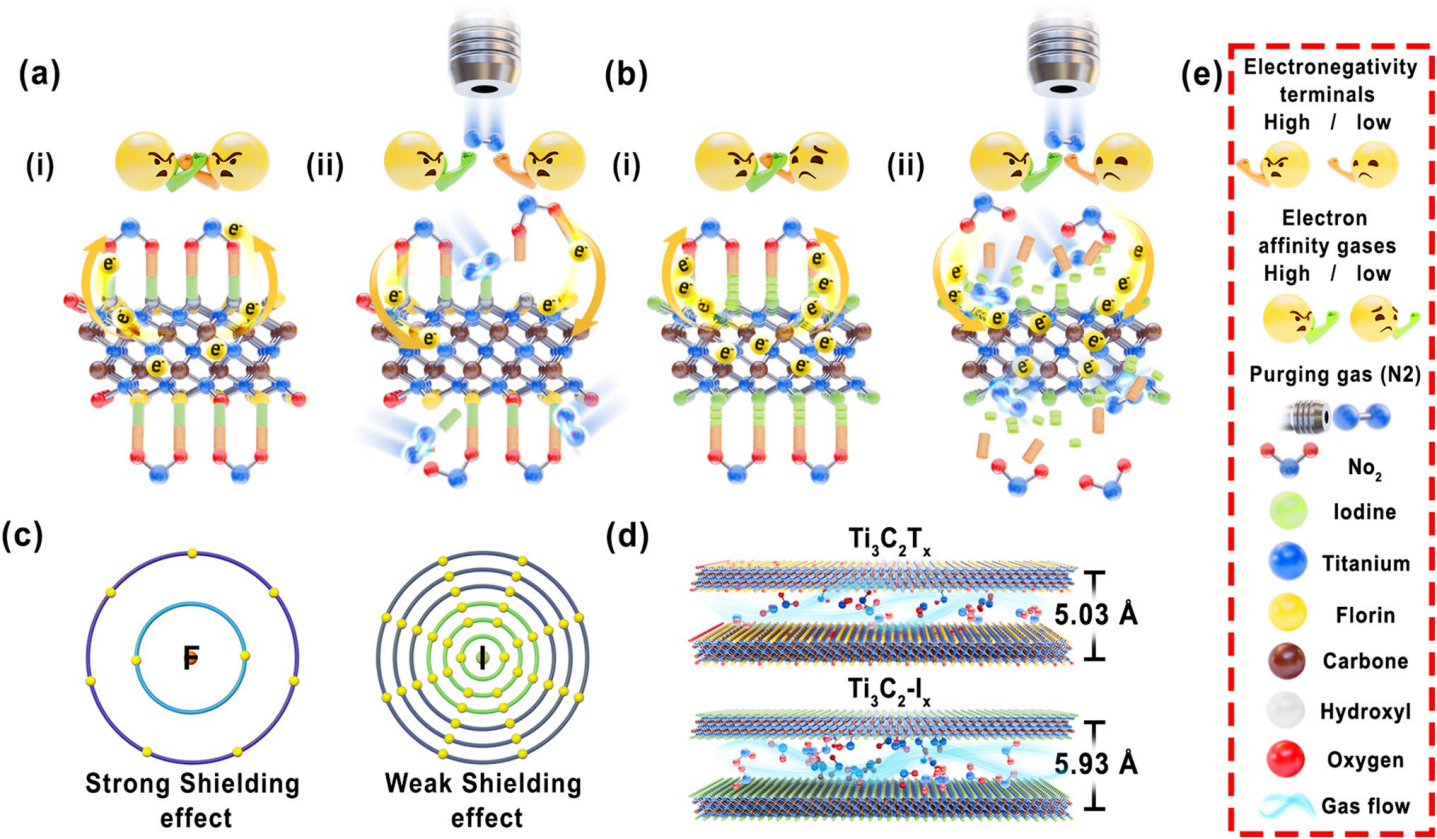
**a, b** Schematics depicting the influence of electronegativity and shielding effects of functional groups (–O/–F/–OH and –I/–Br) on the (i) response and (ii) recovery times when detecting high electron affinity gases **c** Comparison of atomic sizes (–F and –I) indicating differing shielding effects. **d** Enhanced interlayer-spacing of MXene nanosheets achieved via iodine terminals (calculated using Bragg's law) for optimized NO_2_ absorption. **e** Symbol and emoji annotations elucidating essential components within the schematic illustration

The gas sensing mechanism of the developed MXene-based sensors is illustrated in Fig. 5. These sensors operate based on changes in the electrical resistance resulting from the adsorption of gas molecules onto the surface of the sensor [45]. Unlike traditional semiconductor sensors, where the resistance varies based on the oxidizing or reducing gases, MXene-based sensors, owing to their inherent metallic properties, efficiently donate electrons to adsorbed gases regardless of their nature [25]. For instance, in the context of MXene gas sensors, the adsorbed gas molecules (NO_2_ or others) acquire electrons from the MXene surface during adsorption (Eq. 2), leading to an increase in resistance.

2 $${\text{NO}}_{{2}} \left( {{\text{gas}}} \right) \, + {\text{ e}}^{ - } \to {\text{ NO}}_{{2}}^{-} \left( {{\text{ads}}} \right)$$

However, the presence of –O/–OH and –F functional groups, characterized by their elevated electronegativity and robust electron shielding owing to their smaller atomic sizes, hinders rapid electron exchange with gas molecules. This resulted in extended response times for gas-sensing applications (Fig. 5a(i)). Similarly, upon stopping the NO_2_ gas injection and introducing N_2_ gas, a concentration gradient emerged between the densely adsorbed NO_2_^−^_(ads)_ on the MXene surface and the less concentrated NO_2_ gas phase within the chamber. Driven by this gradient, a shift toward equilibrium occurred, leading to the detachment of NO_2_^−^_(ads)_ from the MXene surface. N_2_ gas collisions play a pivotal role in transferring energy, thereby disrupting the adsorption forces and inducing detachment. The resulting NO_2_^−^_(ads)_ shifts to NO_2_ gas, causing desorption, as shown in Eq. (3):

3 $${\text{NO}}_{{2}}^{-} \left( {{\text{gas}}} \right) \, + {\text{N}}_{{2}} \left( {\text{purging gas}} \right) \, \to {\text{ NO}}_{{2}} \left( {{\text{ads}}} \right) \, + {\text{ e}}^{ - }$$

Notably, gases with high electron affinities bonded to the –O/–OH and –F functional groups required more energy for detachment, leading to longer desorption times (Fig. 5a(ii)). Conversely, when the MXene terminals exhibited lower electronegativity and reduced shielding effects owing to larger atomic sizes (Fig. 5c), faster adsorption and desorption occurred (Fig. 5b). Therefore, the excellent selectivity of I–MXene toward NO_2_ is attributed to the high electron affinity of NO_2_, which enables stable bonds for faster charge transport with I–MXene owing to the presence of an unpaired electron in its molecular orbital. In contrast, NH_3_ has a lone pair of electrons available for donation, leading to lower electron affinity. Similarly, acetone and ethanol have lower electron affinities owing to the partial positive charge on the carbon atom caused by the electronegativity difference with the oxygen atom. These lower electron affinities result in weaker electronic interactions with the I–MXene surface, leading to lower sensor responses. Non-polar gases such as H_2_ have symmetrical electron density distributions that weaken their interactions with the MXene surface, resulting in lower sensor responses.

Moreover, the faster response time of I–MXene is attributed to the larger atomic size of iodine, which reduces the shielding effect on the outermost electrons and promotes enhanced electron transfer between gas molecules with high electron affinity and I–MXene. Therefore, the NO_2_ sensor required a shorter response/recovery time (90/122 s) than NH_3_ (90/122 s), ethanol (121/159 s), acetone (114/161 s), and H_2_ (147/142 s) (Figs. 4g and 5b). Furthermore, the exceptional sensitivity and wide detection range arise from the larger atomic size of iodine, which enhances the interlayer spacing for greater gas adsorption (Fig. 5d), and its lower electronegativity, which promotes efficient electron exchange even at low concentrations (50 ppb). These attributes collectively bestow I–MXene with remarkable sensitivity across a broad concentration range (50–200 ppm), distinguishing it from the as-prepared and Br–MXenes. Furthermore, a comparison with previously published studies revealed that I–MXene exhibited remarkable improvements in the recovery time and a wide linear dynamic range, as summarized in Table 1. These findings elucidate the crucial role of terminal functionalization in enhancing the gas-sensing performance of MXenes, providing valuable insights for the development of highly sensitive and faster-responsive gas sensors.

**Table 1:** Comparison of I–MXene with previous studies on functionalized and composite-based MXene gas sensors at room temperature

Material	Type of gas	Concentration (PPM)	Response (%)	Linear range (ppm)	Res/rec time (s)	References
-FOTS functionalized Ti_3_C_2_T_*x*_	Ethanol	120	14.1	5–120	39/139	[25]
CPTMS functionalized Ti_3_C_2_T_*x*_	Ethanol	120	10.1	5–120	120/332	[25]
Hydrocarbon functionalized Ti_3_C_2_T_*x*_	Ethanol	100	14.3	20–500	14.3/37.5	[28]
Oxygen-functionalized MXene	NO_2_	10	13.8	1–10	–/–	[46]
Alkalized organ-like MXene	NH_3_	100	28.87	10–500	1/201	[47]
Ti_3_C_2_T_*x*_/PDS-Cl Composite	H_2_S	5	2	0.5–5	–/–	[48]
Sulfur-doped Ti_3_C_2_T_*x*_	Toluene	50	79.5	1–50	~ 60/ ~ 350	[49]
3D MXene framework	Acetone	5	0.75	0.05–30	90/110	[50]
Ti_3_C_2_T_*x*_/CuO	NO_2_	50	56.99	1–50	13.5/20.9	[51]
Ti_3_C_2_T_*x*_	NO_2_	50	11.91	1–50	40.4/60.9	[51]
Ti_3_C_2_-I	NO_2_	120	14.50	0.05–50	90/105	This work

## Conclusion

In conclusion, we developed a new high-pressure autoclave reactor approach for MXene synthesis and the collection of MXene flakes with a thickness of less than 100 nm over an Si substrate through the interface between two immiscible solvents. Additionally, a unique gas-phase method was employed to eliminate undesired functional groups (–O, –OH, and –F) and introduce I and Br functional groups. The introduction of an iodine termination, characterized by its larger atomic size, reduced shielding effect, and lower electronegativity, played a pivotal role in significantly increasing the surface area, stability, and gas-sensing performance of MXenes. While achieving these advancements, we also identified challenges to improve MXene synthesis, including streamlining the etching process using a Teflon-based high-pressure reactor instead of stainless steel. This adjustment was necessary because HF cannot be treated in a stainless-steel autoclave. In future studies, we intend to optimize the conditions for MXene synthesis using HF-free etchants in a Teflon-based high-pressure reactor. Additionally, we intend to investigate 12 different surface terminations (O, OH, N, NH, NH_2_, S, SH, H, F, Cl, Br, and I) on various carbide-based MXenes to gain valuable insights into the influence of different functional groups and MXene materials on their electrical, environmental, and gas sensing properties. Notably, the proposed methods hold promise for application to other MXene structures and compositions, offering versatility and the potential for further advancements in MXene research across various fields.

## Supplementary Information

Below is the link to the electronic supplementary material.

Supplementary file1 (PDF 3063 kb)
