# Supplementary material for: Tailoring MXene Thickness and Functionalization for Enhanced Room-Temperature Trace NO2 Sensing
Source: Nanomicro Lett. 2024 Jan 12;16:84. doi: 10.1007/s40820-023-01316-x (PMC10786774; doi:10.1007/s40820-023-01316-x)
Supplement: Supplementary file 1 — Supplementary file1 (PDF 3063 kb) [file 40820_2023_1316_MOESM1_ESM.pdf]

Supporting Information for

Tailoring MXene Thickness and Functionalization for Enhanced Room-Temperature Trace NO_2 Sensing

Muhammad Hilal[1,2], Woochul Yang[1,*], Yongha Hwang [2,*], Wanfeng Xie[1,3,*]

[1] Department of Physics, Dongguk University, Seoul, 04620, Republic of Korea

[2] Department of Control and Instrumentation Engineering, Korea University, Sejong 30019, Republic of Korea

[3] College of Microtechnology & Nanotechnology, University-Industry Joint Center for Ocean Observation and Broadband Communication, Qingdao University, Qingdao, 266071, P. R. China

*Correspondence authors. E-mail: wfxie@qdu.edu.cn (Wanfeng Xie), wyang@dongguk.edu (Woochul Yang), hwangyongha@korea.ac.kr (Yongha Hwang)

ORCID: 0000-0002-5477-7988 (Wanfeng Xie), 0000-0003-3726-2269 (Woochul Yang), 0000-0001-7205-5957 (Yongha Hwang)

S1 Series of Experimental Methods for Few-Layer MXene Synthesis: Identifying the Optimal Approach

In this study, a total of 16 experiments were conducted to identify the most suitable condition for preparing few-layer MXene. The experimental conditions, including etching type, stirring time, autoclave temperature, and time, were listed in Tables S1-S16) for each method. SEM analysis for each sample obtained through all the methods was conducted, and the results are presented in Figs. S1 and S2 only for the methods that yielded more distinct and observable interlayer spacing. Fig. S1 specifically highlights the methods that yielded larger interlayer spacing and achieved MXene with a stacked-layer thickness of less than 1 µm. The most promising results were achieved when the MAX phase was stirred in HF (48%) for 36 h and then transferred to a simple autoclave, heated at 150°C for an additional 36 h (Fig. S1f, Fig. S2, and Table S15). This indicates that stirring alone did not significantly etch the A-layer from the MAX phases, resulting in approximately 500 nm thick layer-stacks in the MXene flakes (Fig. S1a and Table SI-1-2). However, transferring the stirred sample to a normal autoclave led to a reduction in thickness to around 150 nm, indicating the importance of applied pressure for effective etchant penetration (Fig. S1f, Fig. S2, and Table S15). Based on these results, a high-pressure stainless-steel autoclave was used, applying both stirring and pressurizing processes simultaneously. This strategic approach further increased etchant penetration between layers and enhanced interaction with a larger number of flakes, thereby achieving uniform layer-stacked thicknesses of about 50 nm throughout all MXene flakes even prior to the exfoliation process (Fig. S1(g-i)), indicating significant removal of the remaining Al. Subsequently, the product underwent 7 hours of tip-sonication and was washed using centrifugation. The sediment collected from the bottom and upper part of the centrifuge tube revealed a stack-layer thickness of about 1~2 µm (Fig. S1j) and <100~600 nm (Fig. 1a and Fig. S3(a-f), respectively. This confirmed that the hybrid HF-Hydrothermal synthesis condition played a critical role in effectively controlling MXene thickness, providing a promising approach for producing few-layer MXene materials with enhanced properties.

Afterward, the sediment collected from the upper part of the centrifuge tube was dispersed in water and diluted to a 10-fold dilution compared to the as-collected sediment concentration (Fig. S4a). The diluted MXene sample was mixed with methanol and transferred dropwise into a beaker containing chloroform solvent, with a Si-wafer positioned at the bottom. Due to the immiscibility between methanol and chloroform created an interface where the few-layer MXene flakes (<100 nm) self-assembled, aligning their size with that of the substrate on the upper surface of the chloroform solvent (Fig. S4b). Subsequently, excess chloroform was removed, and the film level was adjusted to match the substrate. Finally, the substrate was lifted and subjected to a drying process at 100 °C for approximately 30 min to ensure the complete dryness of the MXene's film, revealing a thickness of <50 nm (Fig. 1b and Fig. S3a-c). The main steps involved in this process are illustrated in Schematic. 1 (a-f).

Table S1 Method-1: Different Stirring time

S.No	Sample	solution	Stirrer time	Autoclave	Washing	Freeze-dry	Exfoliation in DMSO
Method-1-1	1g, Ti_3AC_2	20 ml HF	24 h	—	pH ~ 6.0	48 h	2h
Method-1-2	1g, Ti_3AC_2	20 ml HF	36 h	—	pH ~ 6.0	48 h	2h
Method-1-3	1g, Ti_3AC_2	20 ml HF	48 h	—	pH ~ 6.0	48 h	2h

Table S2 Method-2: Different Stirring time

Method-2-1	1g, Ti_3AC_2	6 ml DI, 12ml HCl, & 2 ml HF	24 h	—	pH ~ 6.0	48 h	2h
Method-2-2	1g, Ti_3AC_2	6 ml DI, 12ml HCl, & 2 ml HF	36 h	—	pH ~ 6.0	48 h	2h
Method-2-3	1g, Ti_3AC_2	6 ml DI, 12ml HCl, & 2 ml HF	48 h	—	pH ~ 6.0	48 h	2h

Table S3 Method-3: Different time and 60 °C in the thermal oven

Method-3-1	1g, Ti_3AC_2	1 g LiF in 20 ml HCl	30 min	24 h at 60 °C	pH ~ 6.0	48 h	2h
Method-3-2	1g, Ti_3AC_2	1 g LiF in 20 ml HCl	30 min	36 h at 60 °C	pH ~ 6.0	48 h	2h
Method-3-3	1g, Ti_3AC_2	1 g LiF in 20 ml HCl	30 min	48 h at 60 °C	pH ~ 6.0	48 h	2h

Table S4 Method-4: Different time and 90 °C in the thermal oven

Method-4-1	1g, Ti_3AC_2	1 g LiF in 20 ml HCl	30 min	24 h at 90 °C	pH ~ 6.0	48 h	2h
Method-4-2	1g, Ti_3AC_2	1 g LiF in 20 ml HCl	30 min	36 h at 90 °C	pH ~ 6.0	48 h	2h
Method-4-3	1g, Ti_3AC_2	1 g LiF in 20 ml HCl	30 min	48 h at 90 °C	pH ~ 6.0	48 h	2h

Table S5 Method-5: Different time and 150 °C in the thermal oven

Method-5-1	1g, Ti_3AC_2	1 g LiF in 20 ml HCl	30 min	24 h at 150 °C	pH ~ 6.0	48 h	2h
Method-5-2	1g, Ti_3AC_2	1 g LiF in 20 ml HCl	30 min	36 h at 150 °C	pH ~ 6.0	48 h	2h
Method-5-3	1g, Ti_3AC_2	1 g LiF in 20 ml HCl	30 min	48 h at 150 °C	pH ~ 6.0	48 h	2h

Table S6 Method-6: Different time and 180 °C in the thermal oven

Method-6-1	1g, Ti_3AC_2	1 g LiF in 20 ml HCl	30 min	24 h at 180 °C	pH ~ 6.0	48 h	2h
Method-6-2	1g, Ti_3AC_2	1 g LiF in 20 ml HCl	30 min	36 h at 180 °C	pH ~ 6.0	48 h	2h
Method-6-3	1g, Ti_3AC_2	1 g LiF in 20 ml HCl	30 min	48 h at 180 °C	pH ~ 6.0	48 h	2h

Table S7 Method-7: Different time and 60 °C in the thermal oven

Method-7-1	1g, Ti_3AC_2	1 g NaF in 20 ml HCl	30 min	24 h at 60 °C	pH ~ 6.0	48 h	2h
Method-7-2	1g, Ti_3AC_2	1 g NaF in 20 ml HCl	30 min	36 h at 60 °C	pH ~ 6.0	48 h	2h
Method-7-3	1g, Ti_3AC_2	1 g NaF in 20 ml HCl	30 min	48 h at 60 °C	pH ~ 6.0	48 h	2h

Table S8 Method-8: Different time and 90 °C in the thermal oven

Method-8-1	1g, Ti_3AC_2	1 g NaF in 20 ml HCl	30 min	24 h at 90 °C	pH ~ 6.0	48 h	2h
Method-8-2	1g, Ti_3AC_2	1 g NaF in 20 ml HCl	30 min	36 h at 90 °C	pH ~ 6.0	48 h	2h
Method-8-3	1g, Ti_3AC_2	1 g NaF in 20 ml HCl	30 min	48 h at 90 °C	pH ~ 6.0	48 h	2h

Table S9 Method-9: Different time and 150 °C in the thermal oven

Method-9-1	1g, Ti_3AC_2	1 g NaF in 20 ml HCl	30 min	24 h at 150 °C	pH ~ 6.0	48 h	2h
Method-9-2	1g, Ti_3AC_2	1 g NaF in 20 ml HCl	30 min	36 h at 150 °C	pH ~ 6.0	48 h	2h
Method-9-3	1g, Ti_3AC_2	1 g NaF in 20 ml HCl	30 min	48 h at 150 °C	pH ~ 6.0	48 h	2h

Table S10 Method-10: Different time and 180 °C in the thermal oven

Method-10-1	1g, Ti_3AC_2	1 g NaF in 20 ml HCl	30 min	24 h at 180 °C	pH ~ 6.0	48 h	2h
Method-10-2	1g, Ti_3AC_2	1 g NaF in 20 ml HCl	30 min	36 h at 180 °C	pH ~ 6.0	48 h	2h
Method-10-3	1g, Ti_3AC_2	1 g NaF in 20 ml HCl	30 min	48 h at 180 °C	pH ~ 6.0	48 h	2h

Table S11 Method-11: Different time and 60 °C in the thermal oven

Method-11-1	1g, Ti_3AC_2	5 g NH_4F in 120 ml DI water	30 min	24 h at 60 °C	pH ~ 6.0	48 h	2h
Method-11-2	1g, Ti_3AC_2	5 g NH_4F in 120 ml DI water	30 min	36 h at 60 °C	pH ~ 6.0	48 h	2h
Method-11-3	1g, Ti_3AC_2	5 g NH_4F in 120 ml DI water	30 min	48 h at 60 °C	pH ~ 6.0	48 h	2h

Table S12 Method-12: Different time and 90 °C in the thermal oven

Method-12-1	1g, Ti_3AC_2	5 g NH_4F in 120 ml DI water	30 min	24 h at 90 °C	pH ~ 6.0	48 h	2h
Method-12-2	1g, Ti_3AC_2	5 g NH_4F in 120 ml DI water	30 min	36 h at 90 °C	pH ~ 6.0	48 h	2h
Method-12-3	1g, Ti_3AC_2	5 g NH_4F in 120 ml DI water	30 min	48 h at 90 °C	pH ~ 6.0	48 h	2h

Table S13 Method-13: Different time and 150 °C in the thermal oven

Method-13-1	1g, Ti_3AC_2	5 g NH_4F in 120 ml DI water	30 min	24 h at 150 °C	pH ~ 6.0	48 h	2h
Method-13-2	1g, Ti_3AC_2	5 g NH_4F in 120 ml DI water	30 min	36 h at 150 °C	pH ~ 6.0	48 h	2h
Method-13-3	1g, Ti_3AC_2	5 g NH_4F in 120 ml DI water	30 min	48 h at 150 °C	pH ~ 6.0	48 h	2h

Table S14 Method-14: Different time and 180 °C in the thermal oven

Method-14-1	1g, Ti_3AC_2	5 g NH_4F in 120 ml DI water	30 min	24 h at 180 °C	pH ~ 6.0	48 h	2h
Method-14-2	1g, Ti_3AC_2	5 g NH_4F in 120 ml DI water	30 min	36 h at 180 °C	pH ~ 6.0	48 h	2h
Method-14-3	1g, Ti_3AC_2	5 g NH_4F in 120 ml DI water	30 min	48 h at 180 °C	pH ~ 6.0	48 h	2h

Table S15 Method-15: Combine Method-1-2 and Method-13-2

Sample	solution	Stirrer time	Washing	Added to Solution	Transferred to Teflon Autoclave	Washing	Freeze-dry	Exfoliation in DMSO
1g, Ti_3AC_2	20 ml HF	36 h	pH ~ 6.0	5 g NH_4F in 120 ml DI water	36 h at 150 °C	pH ~ 6.3	48 h	2h

Table S16: Method-16: Combine Method-1-2 and high pressure stainless-steal autoclave reactor

Sample	solution	Stirrer time	Washing	Added to Solution	Transferred to high-pressure Autoclave reactor	Washing	Freeze -dry	Exfoliation in DMSO
1g, Ti_3AC_2	20 ml HF	36 h	pH ~ 6.0	5 g NH_4F in 120 ml DI water	36 h at 150 °C, 200 rpm, and total pressure of 22 Mpa	pH ~ 6.5	48 h	7h

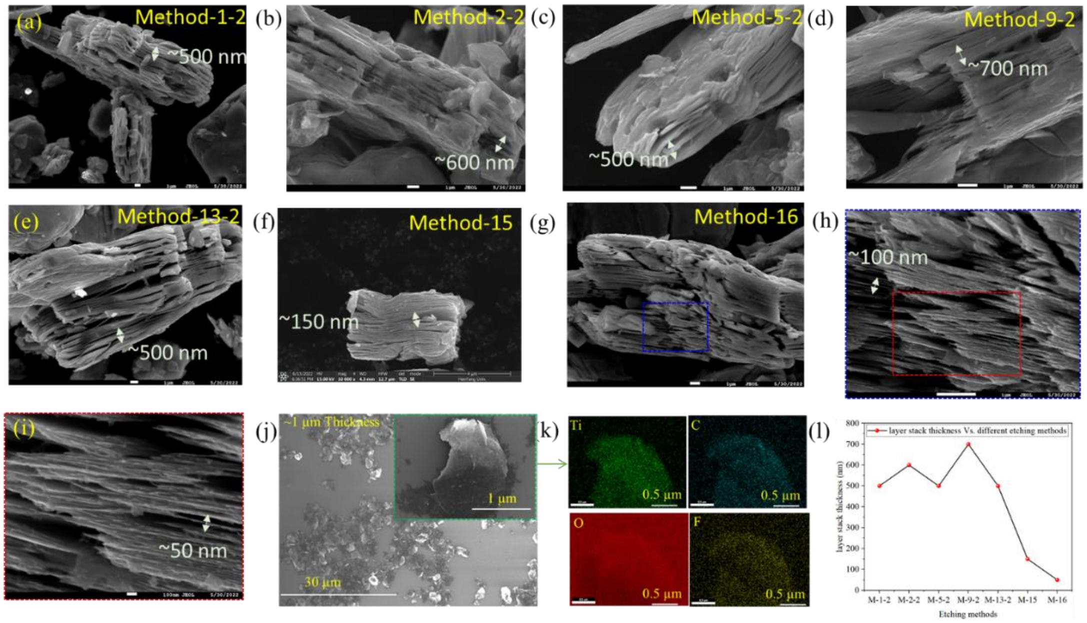

Fig. S1 SEM images showing the layer-stacked thickness of the obtained MXene, with values less than 1 μm. (**k**) Layered-stack thickness obtained using various employed approaches

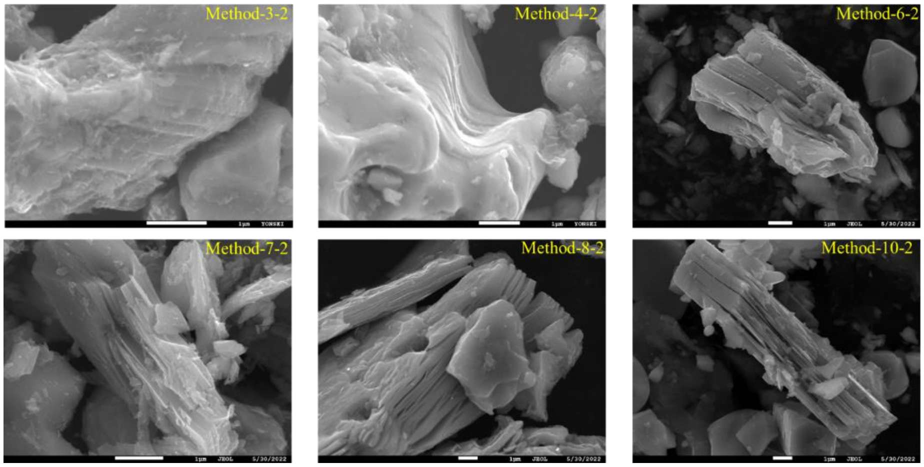

Fig. S2 SEM images of partially etched MXene prepared by stirring Ti_3AlC_2 MAX phase in various etchants for different time

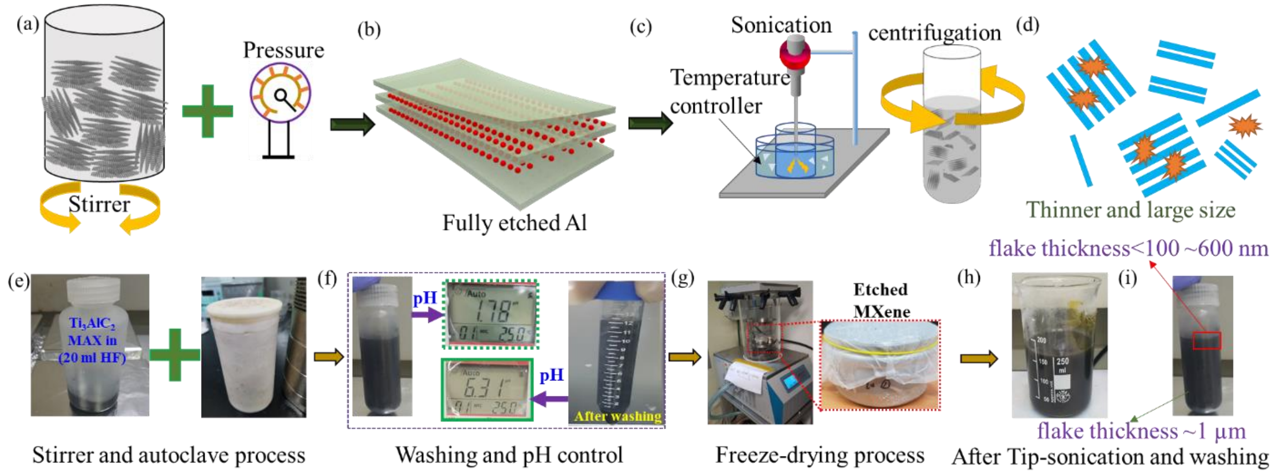

Fig. S3 The process involved in Method-15, combining Method-1-2 and Method-13-2

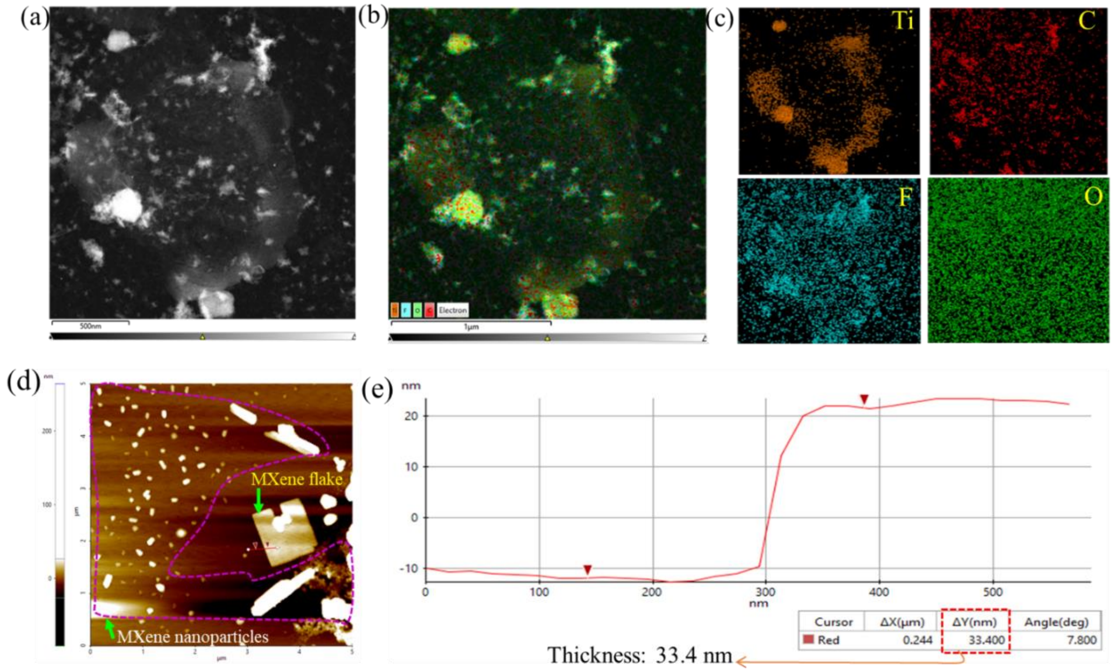

Fig. S4 (**a**) SEM image along with (**b-c**) EDS analysis of fully etched MXene flake, confirming that the white particles are MXene nanoparticles. This is further confirmed by the (**c**) AFM image. (**e**) represents the line profile of the MXene flake to represent its thickness

S2 Thickness measurement of MXene with various thickness

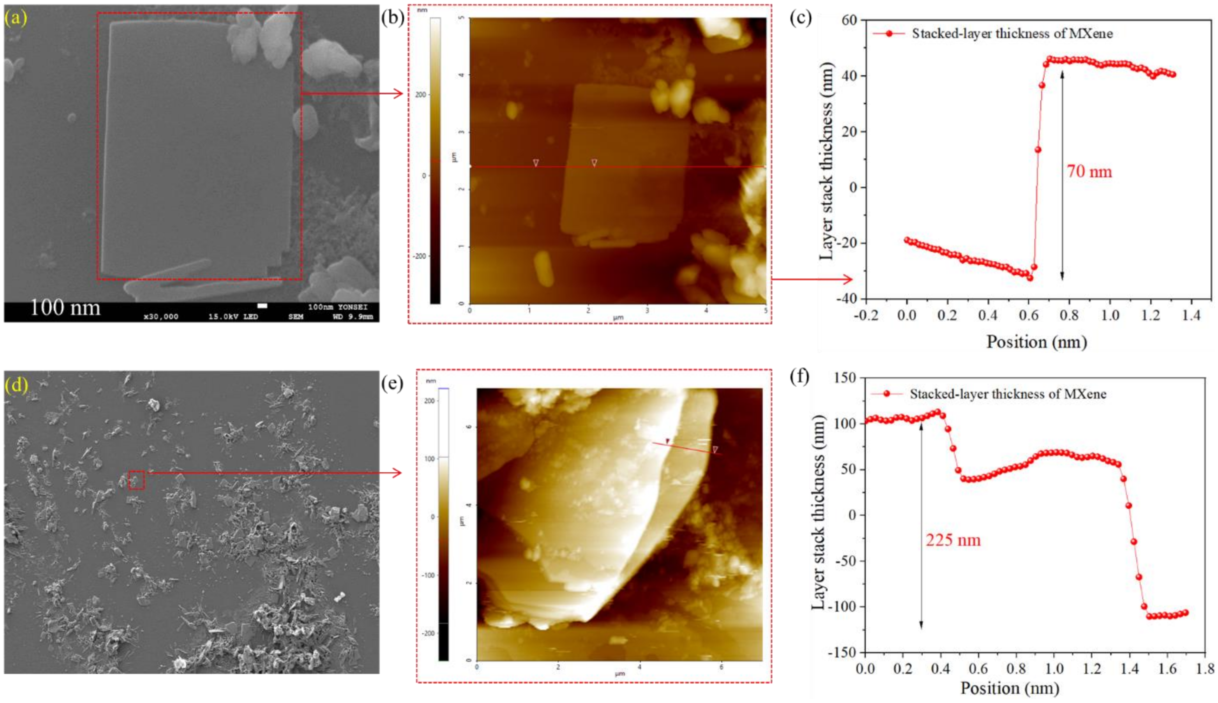

Fig. S5 SEM and AFM images along with thickness profile of (**a-c**) few-layer (<100 nm) self-assembled over Si-wafer and (**d-f**) diluted MXene sample spin-coated over Si-wafer

S3 Synthesis of Cl-MXene via molten salt approach

The Cl-MXene was prepared using a $CuCl_2$ molten salt approach. This method did not involve high-pressure and stirring treatments, resulting in a smaller interlayer separation, as shown in Fig. S6a. The XRD pattern showed a slight shift in the (002) plane from 13.95° to 12.25 degrees (Fig. S6b), confirming reduced interlayer spacing.

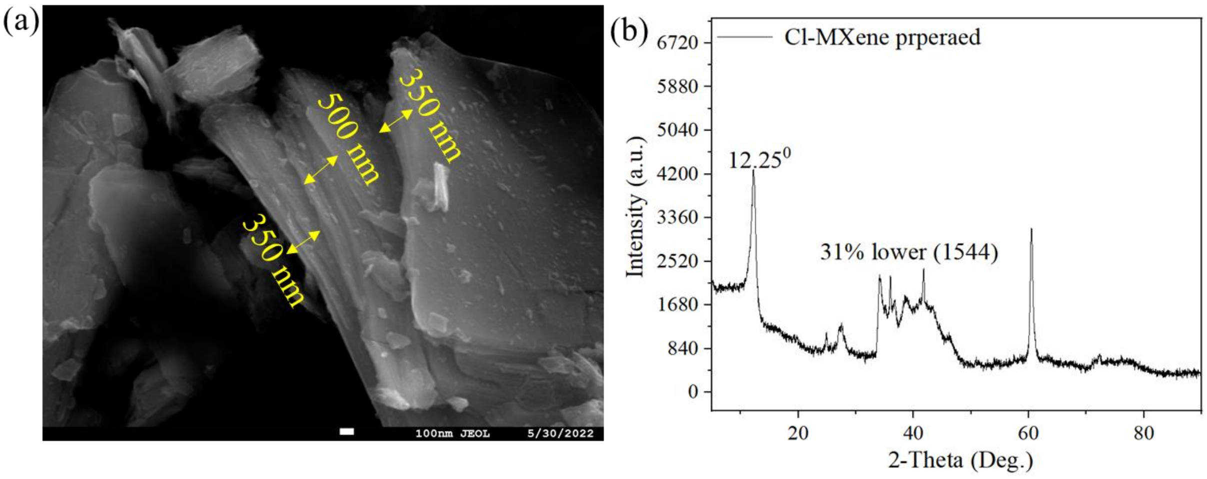

Fig. S6 (**a**) SEM iamge of Cl-MXene prepared via molten salt approach and (**b**) its XRD pattern.

S4 Dilution of MXene solution and its self-assembly over Si-wafer though immiscible solution

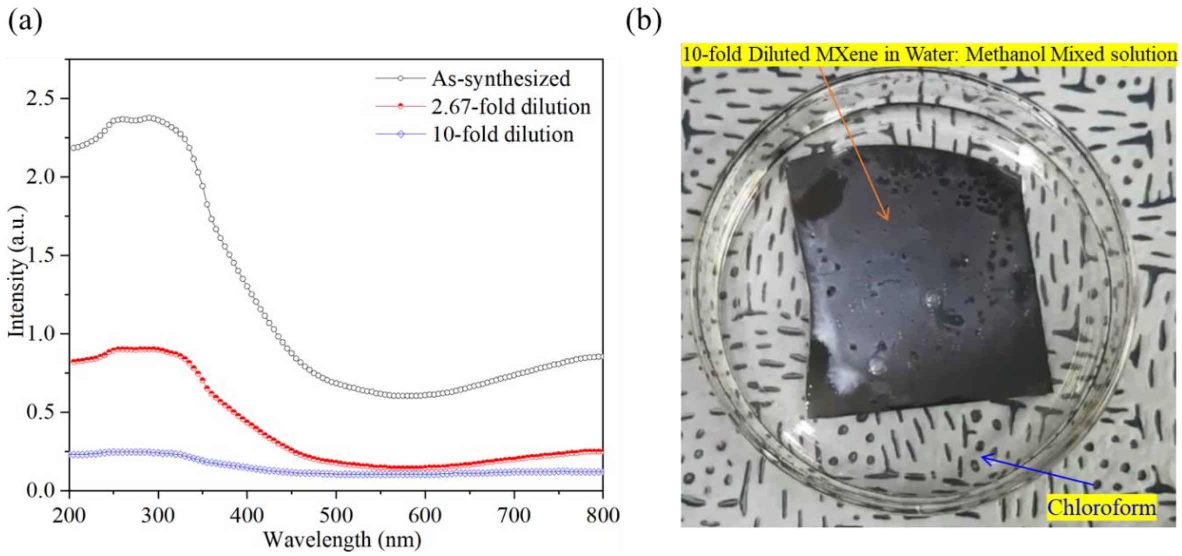

Fig. S7 (**a**) UV-VIS absorbance spectra obtained for an as-synthesized Ti3C2Tx dispersion, as well as 2.67-fold and 10-fold diluted dispersions. (**b**) Formation of an immiscible solution by adding a water: methanol mixture in chloroform for the self-assembly of few-layer MXene over Si-wafer

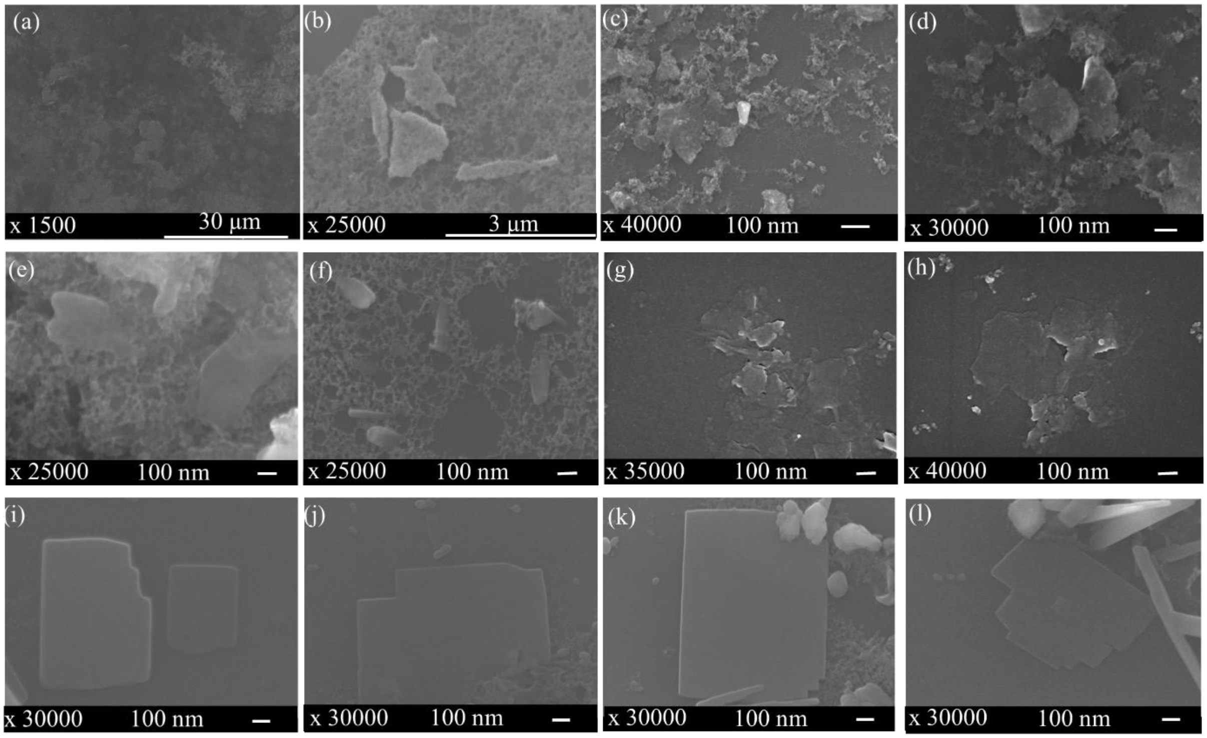

Fig. S8 SEM images of few-layer MXene film obtained via immiscible approach

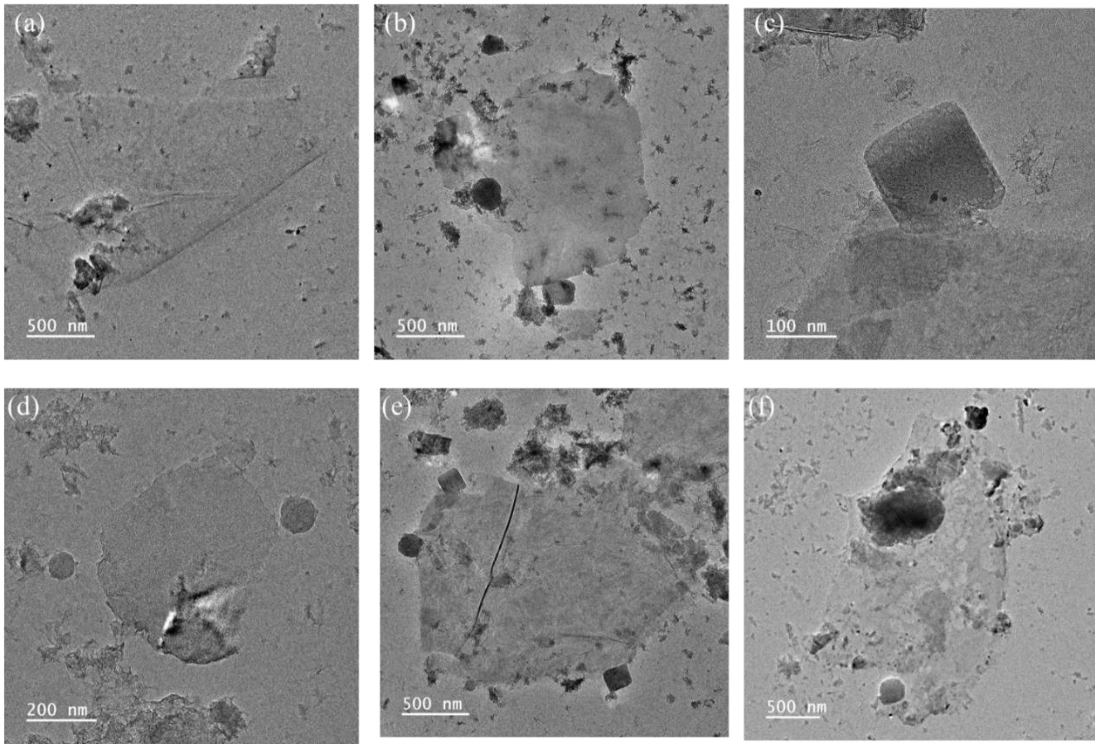

Fig. S9 (**a-f**) TEM images of few-layer MXene film obtained via immiscible approach

S5 Structural and species surface area analysis

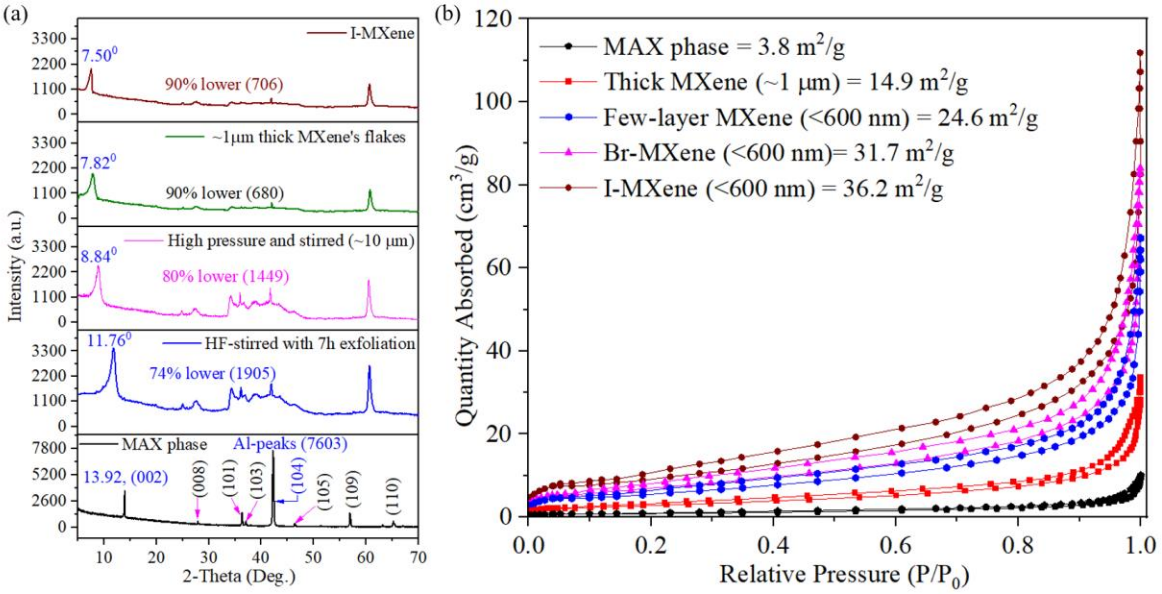

Fig. S10 (**a**) XRD and (**b**) BET analysis of MAX phase, 10 µm thick MXene, 1 µm thick MXene, few-layer MXene, and I-MXene

S6 Hydrophilic and hydrophobic characteristics of as-prepared- and I-MXene

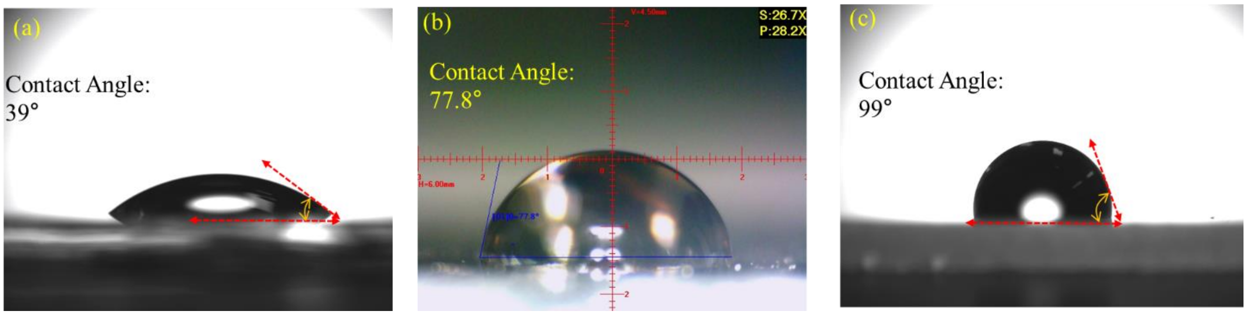

Fig. S11 Contact angle measurement of (**a**) as-prepared-, (**b**) Br-, and (**c**) I-MXene

S7 Role of functionalization on the electrical and environmental properties of MXene

In this comprehensive study, we investigated the resistivity and conductivity of different MXene samples, including as-prepared MXene, Br-MXene, and I-MXene, as given below. Our findings revealed distinct conductivity values for each variant. For I-MXene, the conductivity was measured to be 449.9640029 S/m, while Br-MXene exhibited a conductivity of 394.29067 S/m, and as-prepared MXene showed a conductivity of 341.320226 S/m. Interestingly, recent studies have reported that de-functionalized MXene exhibits higher conductivity compared to functional group-based MXene. This observation can be attributed to the trapping of electrons by functional groups in the MXene channel (Ti3C2). Upon de-functionalization, these trapped electrons are released and reintegrated into the Ti3C2 structure, resulting in higher conductivity. Consequently, MXenes with high electronegative functional groups, such as -O, -OH, and -F, are expected to exhibit lower conductivity due to their higher tendency to trap electrons from the Ti3C2 MXene. Consistent with these findings, our study demonstrated that MXenes with -O, -OH, and -F-based functional groups exhibited lower conductivity compared to MXenes with lower electronegative terminal groups such as -I and Br-. This correlation highlights the influence of the terminal group's electronegativity on electron trapping and conductivity in MXene materials. These results not only contribute to a better understanding of MXene properties but also pave the way for tailoring MXenes with specific functional groups to achieve desired electrical characteristics for diverse applications in electronics, energy storage, and sensors.

Conductivity calculation

For I-MXene:

Resistance (R) = 3705 Ω

Length (L) = 0.5 cm = 0.005 m

Width (W) = 0.3 cm = 0.003 m

Thickness (T) = 0.6 µm = 0.0000006 m

Area (A) = W * T = $1.8E^{-9}$ m^2

Resistivity (ρ) = R * (A / L) = 0.00133344 Ω .m

Conductivity (σ) = 1 / ρ = 749.9400048 S/m

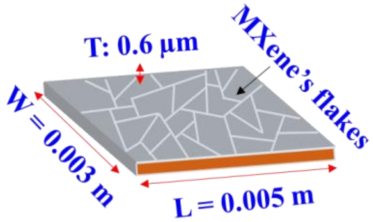

Schematic S1 Conductivity measurement for MXene film over Si-wafer.

For Br-MXene

Resistance (R) = 4227 Ω

Resistivity (ρ) = R * (A / L) = 0.00152172 Ω .m

Conductivity (σ) = 1 / ρ = 657.1511185 S/m

For As-prepared-MXene

Resistance (R) = 4883 Ω

Resistivity (ρ) = R * (A / L) = 0.00175788 Ω .m

Conductivity (σ) = 1 / ρ = 568.8670444 S/m

Moreover, the stability of both I-MXene and as-prepared MXene was evaluated in ambient and aqueous environments. In the ambient environment, I-MXene exhibited reasonable stability for up to 80 days, with a 176% change in resistance. However, beyond the 150th day, a substantial increase in resistance exceeding 203,882% was observed, indicating significant instability. On the other hand, as-prepared MXene showed an extremely high change in resistance, reaching 99,613% by the 90th day, with no response observed beyond 110 days. In the aqueous environment, I-MXene exhibited an initial increase in resistance of about 172% during the first week, followed by more dramatic changes of 25,762% and 255,665% in the second and third weeks, respectively. Eventually, in the fourth week, no response was detected, suggesting complete oxidation. On the other hand, as-prepared MXene demonstrated significant increases in resistance, with a change in response reaching 16,903% in the first week and 186,385% in the second week. However, no response was observed in the third week, indicating weak oxidation. Notably, the high hydrophobicity of I-MXene contributed to its enhanced oxidation stability compared to as-prepared MXene in both ambient and aqueous environments, offering a promising solution for improving the long-term performance and reliability of MXene-based materials.

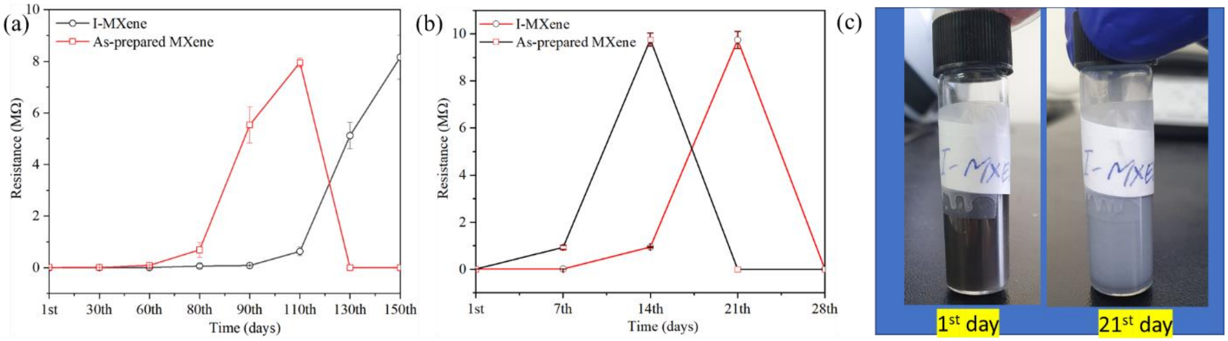

Fig. S12 The resistance variation of both As-prepared and I-MXene over time (days) when stored in (**a**) ambient environment and (**b**) aqueous environment

S8 Elemental analysis and functional groups confirmation

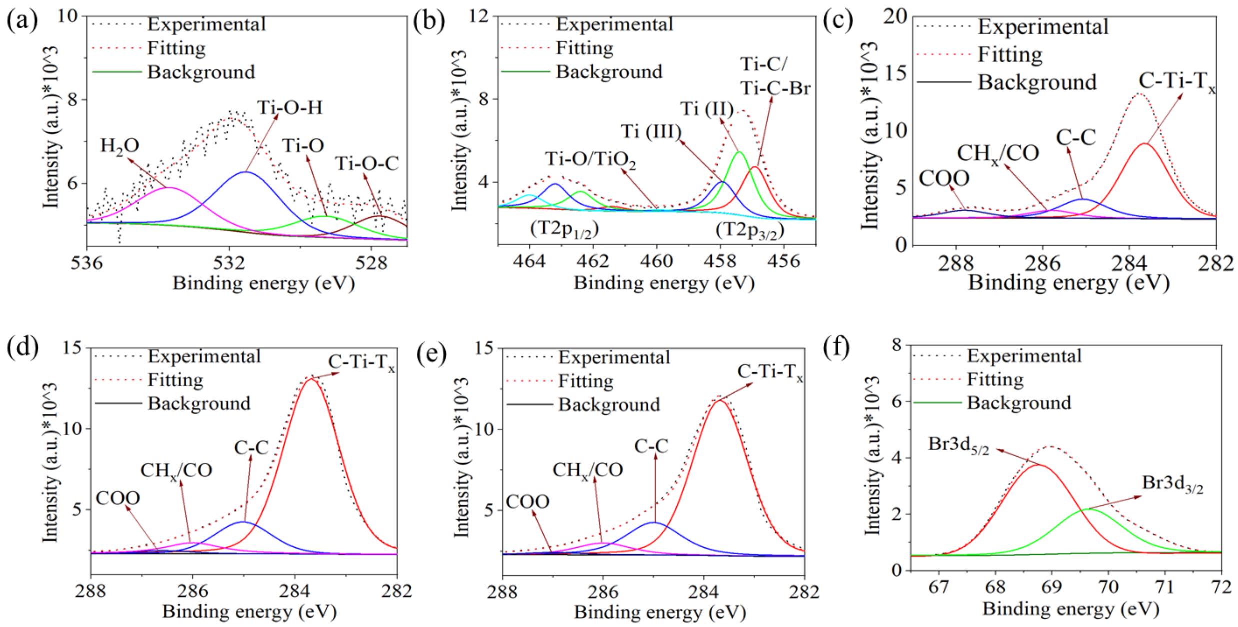

Fig. S13 Illustrates the high-resolution (**a**) O1s and (**b**) Ti2p spectra of Br-MXene. The C1s spectra of (**c**) as-prepared-MXene, (**d**) I-MXene and (**e**) Br-MXene. (**f**) The Br3d spectrum of Br-MXene

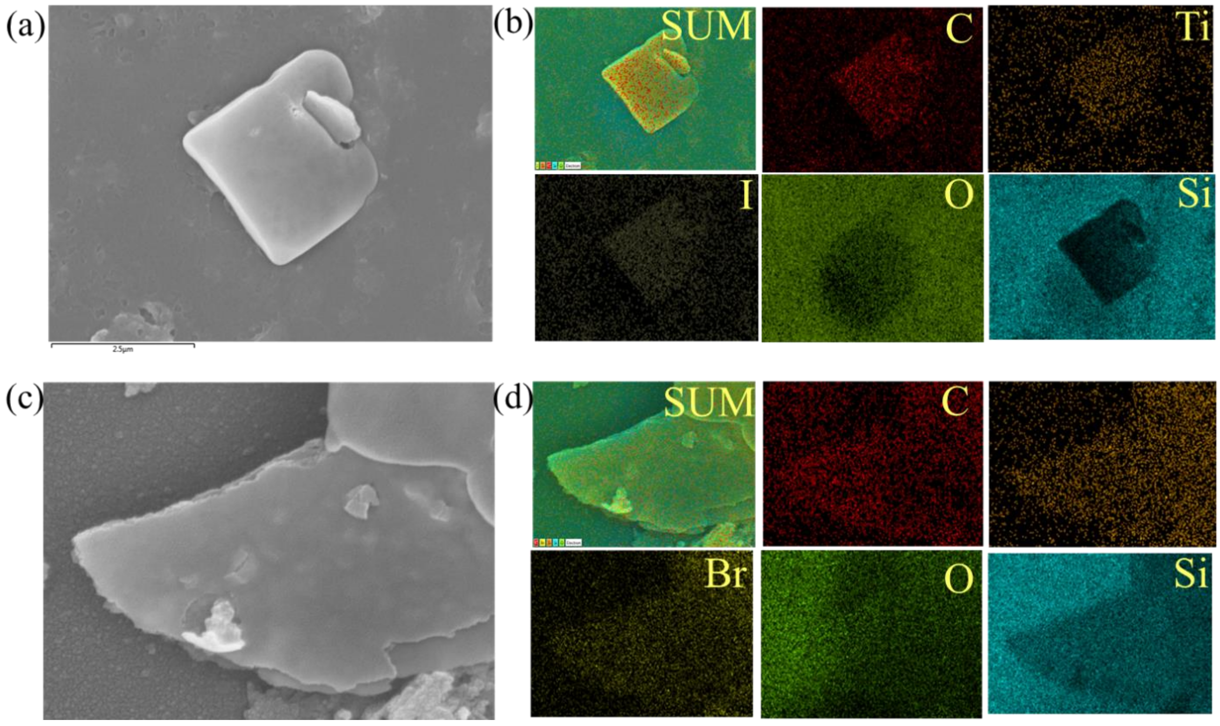

Fig. S14 (a, c) SEM images of MXene, with layer-stacked thickness of 1 μm, which were successfully functionalized with (**b**) Iodine and (**d**) Br

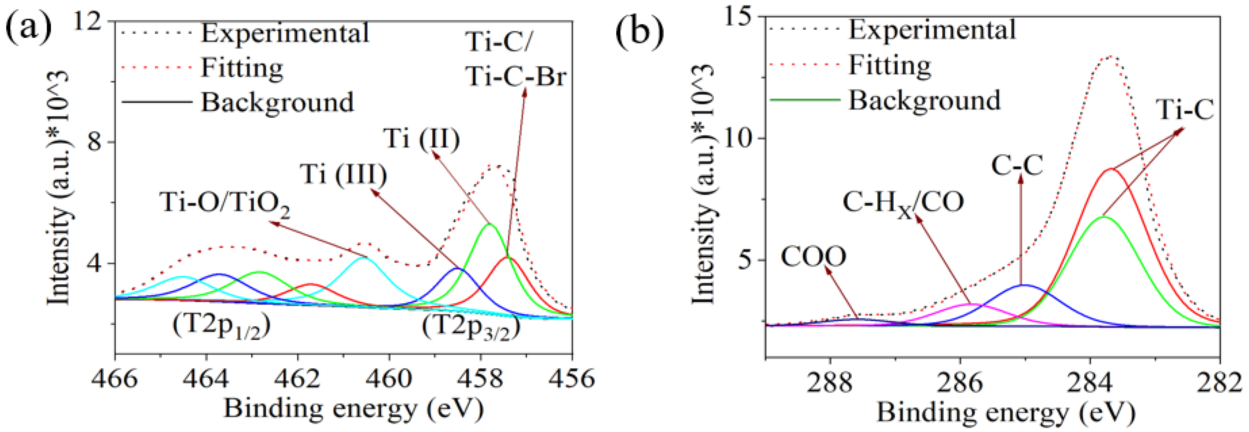

Fig. 15 Depicts the high-resolution (**a**) Ti 2p and (**b**) C 1s spectra of Br-MXene

S9 Gas Sensing Device Preparation and Measurement setup

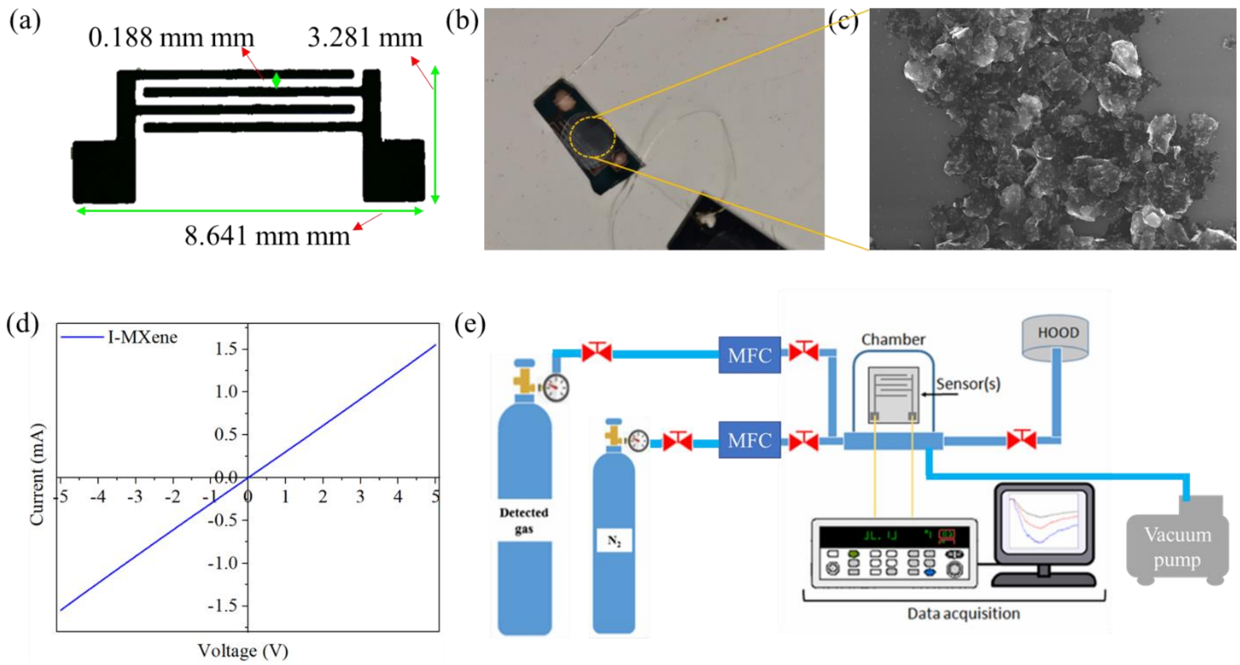

Fig. S16 (**a**) Dimension measurements of the interdigitated electrode, (**b**) an optical image of the sensing device created by (**c**) drop-casting fully etched MXene flakes, (**d**) the I-V curve of the sensing device, indicating an ohmic contact, and (**e**) the gas sensing setup used in this study

S10 Role of stacked-layered and flake thickness on the gas sensing performance of MXene

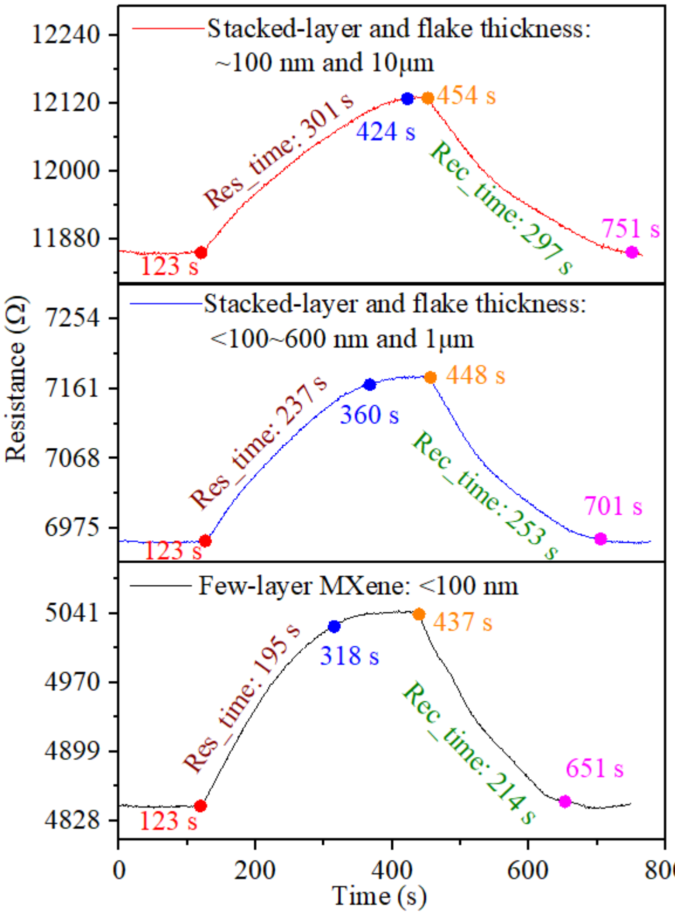

Fig. S17 Variation in gas sensor's resistance along with response/recovery time

S11 Role of functionalization on the gas sensing performance of MXene

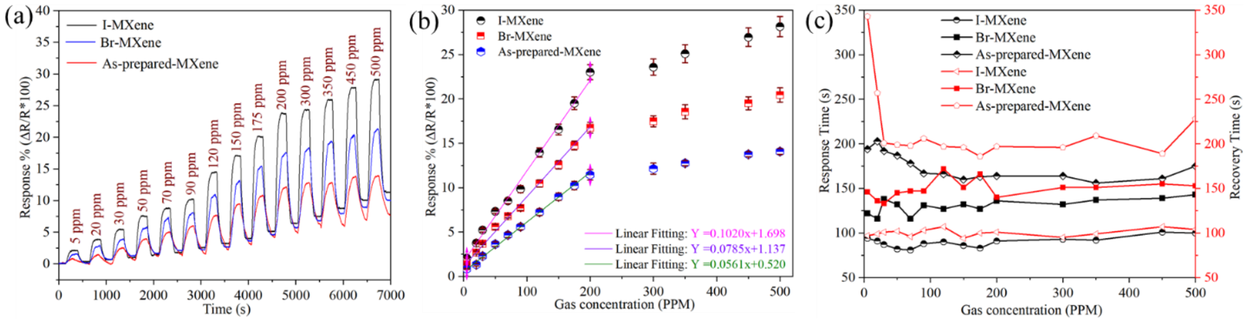

Fig. S18 (**a**) Dynamic response curves to NO_2 concentrations (5-500 ppm) with corresponding (**b**) response versus NO_2 concentration plots. (**c**) Response with recovery time of As-prepared-, Br-, and I-MXene based sensors

S12 Selectivity of Br-MXene and Effect of Humidity on NO_2 Sensing Performance of I-MXene

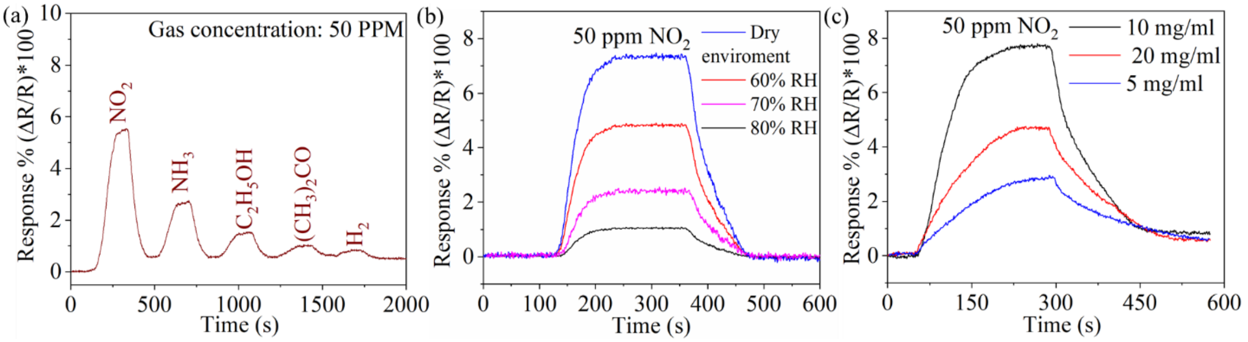

Fig. S19 (a) Selectivity performance of Br-MXene, (b) gas sensing performance of I-MXene toward a 50-ppm concentration of NO_2 at various humidity levels, (c) Dose-Response relationship of MXene gas sensor exposed to 50 ppm NO_2 concentration.

S13 Role of functional groups on oxidation stability and gas sensing performance of MXene

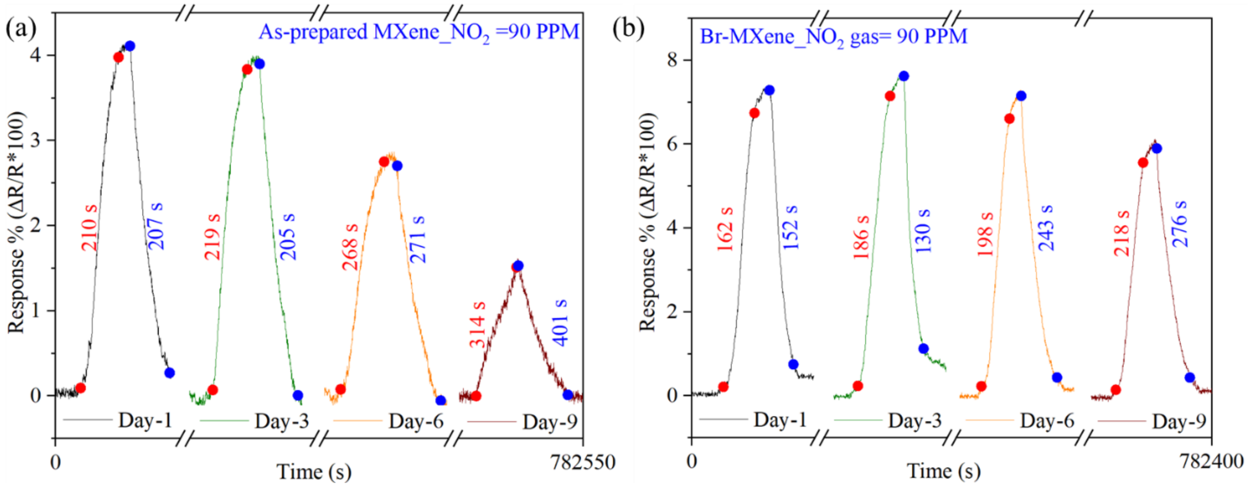

Fig. S20 Stability performances of (**a**) As-prepared- and (**b**) Br-MXene towards four pulses of NO2 conducted over a 9-day period

S14 Gas sensing performances of Cl-MXene

SEM and XRD patterns (Fig. S6) confirm that Cl-MXene retained a higher Al content, as indicated by a slight downward shift in the (002) peak. Consequently, Cl-MXene exhibited higher resistance (15.5 KΩ) and limited response to 50 ppm of NO_2 (1.2%) when compared to our as-prepared multi-layered MXene. The improved performance of the as-prepared MXene is attributed to its larger interlayer separation achieved through greater Al etching.

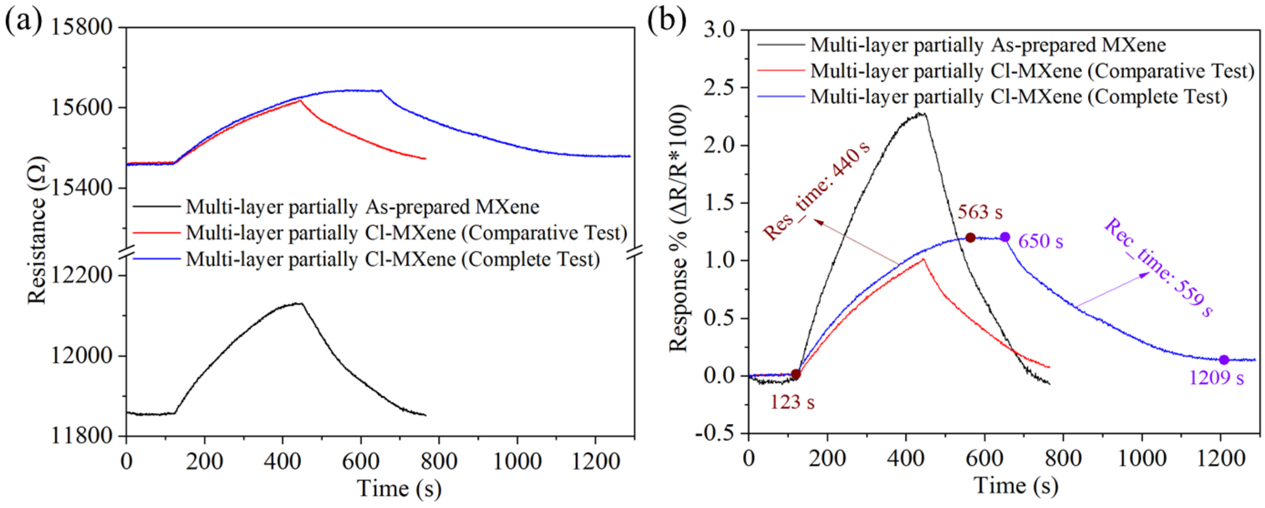

Fig. S21 Gas sensing response of Cl-MXene, multi-layered MXene and their comparison
